# Gut microbiota depletion delays somatic peripheral nerve development and impairs neuromuscular junction maturation

**DOI:** 10.1080/19490976.2024.2363015

**Published:** 2024-06-07

**Authors:** Matilde Cescon, Giovanna Gambarotta, Sonia Calabrò, Chiara Cicconetti, Francesca Anselmi, Svenja Kankowski, Luisa Lang, Marijana Basic, Andre Bleich, Silvia Bolsega, Matthias Steglich, Salvatore Oliviero, Stefania Raimondo, Dario Bizzotto, Kirsten Haastert-Talini, Giulia Ronchi

**Affiliations:** a Department of Molecular Medicine, University of Padova, Padova, Italy; b Department of Clinical and Biological Sciences & Neuroscience Institute Cavalieri Ottolenghi (NICO), University of Torino, Orbassano, Italy; c Department of Life Sciences and Systems Biology, University of Torino, Torino, Italy; d Institute of Neuroanatomy and Cell Biology, Hannover Medical School, Hannover, Lower-Saxony, Germany; e Institute for Laboratory Animal Science and Central Animal Facility, Hannover Medical School, Hannover, Lower-Saxony, Germany; f Research Core Unit Genomics, Hannover Medical School, Hannover, Lower-Saxony, Germany; g Centre for Systems Neuroscience (ZSN), Hannover Medical School, Hannover, Lower-Saxony, Germany; h Department of Biology, University of Padova, Padova, Italy

**Keywords:** Germ-free mice, gnotobiotic mice, peripheral nerve development, skeletal muscle, myelin, Schwann cells, microbiota

## Abstract

Gut microbiota is responsible for essential functions in human health. Several communication axes between gut microbiota and other organs via neural, endocrine, and immune pathways have been described, and perturbation of gut microbiota composition has been implicated in the onset and progression of an emerging number of diseases. Here, we analyzed peripheral nerves, dorsal root ganglia (DRG), and skeletal muscles of neonatal and young adult mice with the following gut microbiota status: a) germ-free (GF), b) gnotobiotic, selectively colonized with 12 specific gut bacterial strains (Oligo-Mouse-Microbiota, OMM12), or c) natural complex gut microbiota (CGM). Stereological and morphometric analyses revealed that the absence of gut microbiota impairs the development of somatic median nerves, resulting in smaller diameter and hypermyelinated axons, as well as in smaller unmyelinated fibers. Accordingly, DRG and sciatic nerve transcriptomic analyses highlighted a panel of differentially expressed developmental and myelination genes. Interestingly, the type III isoform of Neuregulin1 (NRG1), known to be a neuronal signal essential for Schwann cell myelination, was overexpressed in young adult GF mice, with consequent overexpression of the transcription factor Early Growth Response 2 (*Egr2*), a fundamental gene expressed by Schwann cells at the onset of myelination. Finally, GF status resulted in histologically atrophic skeletal muscles, impaired formation of neuromuscular junctions, and deregulated expression of related genes. In conclusion, we demonstrate for the first time a gut microbiota regulatory impact on proper development of the somatic peripheral nervous system and its functional connection to skeletal muscles, thus suggesting the existence of a novel ‘Gut Microbiota-Peripheral Nervous System-axis.’

## Introduction

Gut microbiota consists of a large variety of symbiotic microorganisms (e.g., bacteria, viruses, fungi, and single-celled eukaryotes), who co-evolved with the host to form a mutually beneficial relationship.^1^ The microbial colonization of the human gut begins immediately at birth and it is influenced by numerous factors including mode of delivery (vaginal or cesarean section), feeding regime (breastfeeding or formula feeding), maternal diet/weight, probiotic and prebiotic use, and antibiotic exposure. By the age of 2.5 years, the human infant microbiota starts resembling that of adults in terms of composition, diversity, and functionality. Throughout life, many other factors such as geographical provenance, dietary habits, physical exercise, antibiotic use, age, stress, hormones, circadian rhythms and pathologies in and outside the gastrointestinal tract continue to influence and modify the gut microbiota composition.^2^ The gut microbiota has been established to be responsible for healthy conditions or pathologies of mammalian organisms. Furthermore, it is not only the post-natal gut microbiota that is important, evidence exists that also the maternal microbiota has an impact on the neurodevelopment of the embryo.^3^ The gut microbiota processes dietary nutrients converting them into microbial-derived metabolites that can communicate with organs and tissues throughout the body, establishing intricate connections known as ‘gut-organ axes’. This communication can occur through various systemic signaling, including humoral, endocrine, metabolic and immune pathways, highlighting the complex interplay between the gut and peripheral organs.^4^

Recent studies have provided data on how gut microbiota influences other organs outside the actual gastrointestinal tract, leading to the definition of the ‘gut-kidney axis’,^5^ ‘gut-liver axis’,^6^ ‘gut-muscle axis’,^7^ ‘gut-lung axis’,^8^ ‘gut-heart axis’^9^ and others. Furthermore, the ‘gut-brain axis’ has been extensively explored.^1^ Perturbance of the gut microbiota is implicated as a considerable factor in the development of a growing list of neurodegenerative diseases, neurological pathologies, and psychiatric disorders.^10–15^ Studying the reciprocal impact between the gut microbiota and the health of the central nervous system (CNS) is a growing field in science, and implications are about to derive for new therapeutic approaches.^1^

Microbiota-derived metabolites and other products, such as peptides and neuroactive substances, affect CNS development and function^1^ and are expected to similarly impact on the peripheral nervous system (PNS).^16^ Peripheral nerves originate from the neuroectoderm and elongate with the growth and development of mesodermal somites, which further develop into bone and muscle tissue. Schwann cells, glial cells of the PNS, and dorsal root ganglion neurons, develop from the ectodermal neural crests; peripheral nerves and skeletal muscle or sensory target tissue build an interdependent unit. Neurons and their processes in the PNS belong to somatomotor, somatosensory, visceromotor, and viscerosensory systems.

To the best of our knowledge, no studies have reported whether gut microbiota regulates the development or function of somatic PNS in healthy/physiological conditions. While the impact of gut microbiota on the development of the enteric nervous system (a branch of the PNS located within the walls of the gastrointestinal tract) and its functional target tissues appears to be clear,^16–18^ the impact of gut microbiota on the communication and functional dependence of somatic nerves and their target tissues remains to be revealed. Based on the presented results, we contribute new knowledge to fill this gap.

The most acknowledged approach for determining how the gut microbiota may influence the development or function of a specific tissue or organ is to utilize a germ-free animal model.^19^ Germ-free (GF) mice are bred in isolators completely preventing their contact with environmental and nutritional microorganisms and optimally enabling the study of gut microbiota-dependent developmental processes. Such a model provides an excellent starting point for inoculating a defined and strictly controlled microbiota to unravel the details of this interdependency.^20^ One major example of a well-controlled microbiota model is the Oligo-Mouse-Microbiota 12 (OMM12) model, a mouse colony established in GF status, developed and employed to understand the impact of microbiota on gnotobiotic status with long-term stability.^21^ The OMM12 microbiota includes 12 genome-characterized strains isolated from mice, representing five bacterial phyla naturally present in the murine gastrointestinal tract (Firmicutes, Bacteroidetes, Verrucomicrobia, Actinobacteria, and Proteobacteria).^11^ GF and OMM12 mice represent models completely different from mice naturally colonized with complex gut microbiota (CGM), that represents natural microbiota with hundreds of non-defined bacterial species, but potentially also viral, fungal, archaeal and protozoan species.^22^ Mouse models are, however, much less standardized with regard to the non-bacterial species that could also contribute modulatory molecules and metabolites and further complicate the definition of a potential “gut – somatic nervous system axis”.

In the current study, we aimed to reveal any potential link between gut microbiota and the peripheral components of the somatic nervous system. Therefore, we focused our interest on evaluating the gene expression profiles of peripheral nerves and dorsal root ganglia, as well as the histomorphometry of peripheral nerves, skeletal muscles, and neuromuscular junctions. We comparatively analyzed tissue samples from neonatal and young adult GF, OMM12, and specific pathogen-free mice characterized by a complex gut microbiota (CGM).

## Results

### The gut microbiota analysis from OMM12 and CGM mice confirms distinct taxonomic composition and distinct differences in functional gene annotation in KEGG analysis

Taxonomic profiling of OMM12 and CGM gut communities was carried out by a shallow shotgun metagenomic sequencing of fecal pellets collected from age-matched OMM12 and CGM-colonized animals from stable breeding colonies. By plotting principal coordinates at species level the clear compositional difference between OMM12 and CGM groups can be visualized (Figure S1A). Additionally, from the distance matrix it is visible that CGM samples are showing higher individual differences than samples from the OMM12 group (Figure S1A). The compositional difference on taxonomic level was shown using a bar chart showing the top 10 abundant genera in CGM colonized mice and species distribution of OMM12 members (Figure S1B). Using additional bioinformatic analysis the reads of OMM12 colonized mice acquired by shallow shotgun sequencing were additionally blasted against the genomes of OMM12 species to group highly similar sequences together. On the species level all members of the OMM12 community except *Bifidobacterium longum subsp. animalis* YL2 have been confirmed with shallow metagenome sequencing and OMM12 bacterium-specific 16S rRNA gene copy number assays (Figure S1B and S1C). Furthermore, no distinct taxa have been identified that would point to the microbial contamination of the OMM12 community. The low abundance of YL2 is in line with compositional status of the OMM12 community described in other studies.^23^ In addition, OMM12 bacterium-specific 16S rRNA gene copy number analysis has been used to detect the presence of OMM12 representatives in CGM colonized mice. With the analysis, seven members of the OMM12 community have also been detectable in the CGM community including *Limosilactobacillus reuteri* I49, *Muribaculum intestinale* YL27, *Acutalibacter muris* KB18, *Akkermansia muciniphila* YL44, *Enterococcus faecalis* KB1, *Bifidobacterium longum subsp.animalis* YL2 and *Turicimonas muris* YL45 (Figure S1D). Furthermore, community functional annotation based on the KEGG database showed the difference in number of genes annotated to six classes on level 1 (metabolism, human diseases, organismal systems, cellular processes, genetic information processing, and environmental information processing). The major difference in gene annotation between OMM12 and CGM group was demonstrated for the genes annotated to metabolic pathways, as shown in the heat map analysis (Figure S1E). At the level 2 annotation, in classes organismal systems and human diseases, OMM12 and CGM community showed differences in genes annotated to nervous system, suggesting a direct link between bacterial genes and nervous system (Figure S1F).

### Microbiota depletion alters the transcriptome profiles in sciatic nerves and dorsal root ganglia

To investigate whether gut microbiota can affect the development and maturation of the peripheral nervous system (PNS), we first focused on molecular changes occurring in the sciatic nerves and dorsal root ganglia (DRG) collected from CGM, GF, and OMM12 mice. With this aim, we performed transcriptome analysis by RNA sequencing (RNA-seq) on tissues from neonatal (12–14 days old) and young adult (63–67 days old) mice.

#### Findings from neonatal animals

**Figure 1.**
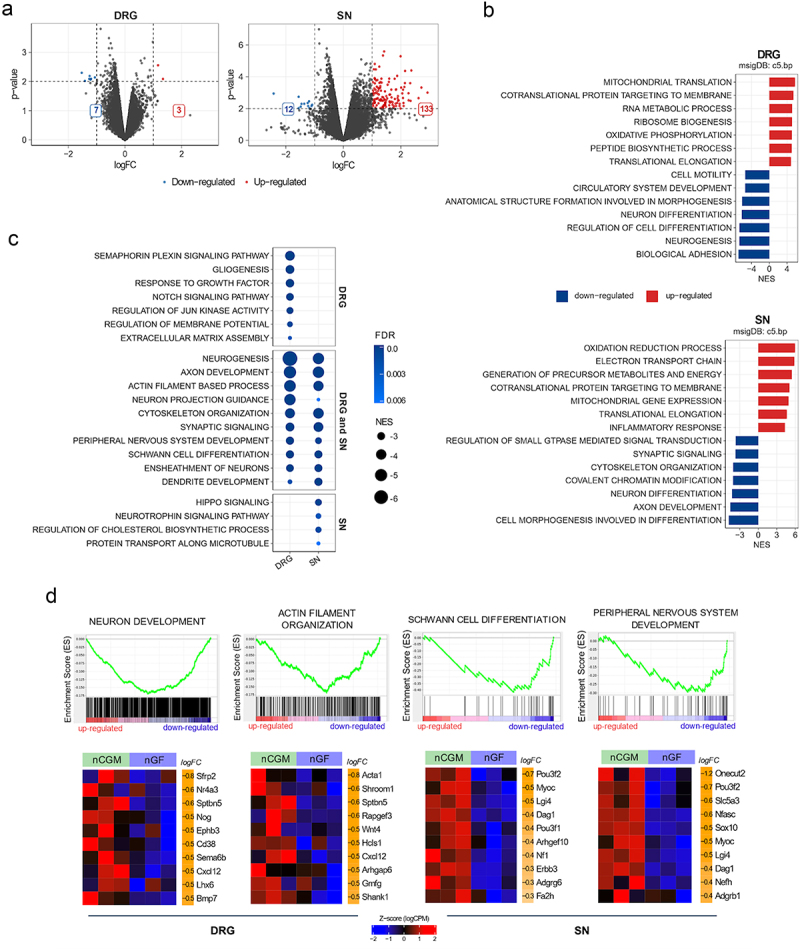
Transcriptomic analysis of neonatal GF compared to CGM dorsal root ganglia (DRG) and sciatic nerves (SN). (a) Volcano plots showing differentially expressed genes in neonatal DRG and sciatic nerve (GF n = 3; CGM n = 3). Genes with a | log_2_FC | > 1 and with -log_10_(p-value) > 2 are highlighted in red and blue respectively. (b) Top 7 positively correlated (red) and negatively correlated (blue) with GF phenotype GO biological processes (FDR <0.01) from GSEA of neonatal DRG and sciatic nerve. The reported value is the Normalized Enrichment Score (NES). (c) Dot plot showing a list of selected negatively enriched GO gene sets related to peripheral nervous system development, maturation and function. Dot size relates to NES value; dot color represents the FDR. The biological processes are divided into three groups: significantly correlated in DRG only (top), sciatic nerve only (bottom) and both (center). (d) GSEA plots of interesting negatively enriched GO gene sets found in DRG. Each GSEA plot is associated with a heatmap reporting the expression value as normalized log (CPM) of the top 10 genes in the selected gene set ordered according to logFC in neonatal CGM and GF. The log_2_FC value is reported in the rightmost column of each heatmap (FDR: false discovery rate; GO: gene ontology).

Ten genes were found to be differentially expressed (| log_2_FC | >1; *p* < 0.01) in neonatal DRG samples from GF compared to CGM mice (three upregulated and seven downregulated genes, Figure 1a). In neonatal sciatic nerve samples from GF compared to CGM mice, 145 genes were found to be differentially expressed (133 upregulated and 12 downregulated genes; Figure 1a).

To understand the biological processes affected by the absence of gut microbiota in neonatal mice, Gene Set Enrichment Analysis (GSEA) of the differentially expressed genes (DEGs) was performed using the Gene Ontology (GO) database. Gene sets involved in mitochondrial function, redox reaction, ribosome biogenesis, and RNA metabolism (Figure 1b, upper panel) were positively correlated with GF status in the DRG. Meanwhile, mitochondrial function and immune system activation were positively correlated with GF status in the sciatic nerve (Figure 1b, lower panel). Biological processes related to cell motility, neurogenesis, and circulatory system development were negatively enriched in the DRG (Figure 1b, upper panel). Neuron differentiation, function, and cytoskeleton organization were negatively enriched in the sciatic nerve (Figure 1b, lower panel). Interestingly, gene sets associated with myelin sheath formation, Schwann cell/glial cell differentiation, neuron/axon development and function, microtubule organization, and extracellular matrix assembly were negatively correlated with GF status (Figure 1c), pointing to impaired development of the PNS in GF compared to CGM mice. This indication was further confirmed by the significant negative enrichment of the gene set “PNS development” in both the DRG and sciatic nerve (Figure 1c). Examples of the most representative GSEA plots and relative heat maps showing the top 10 differentially expressed genes according to logFC are shown in Figure 1d.

By comparing neonatal OMM12 with CGM DRG, we detected a total of 33 DEGs in OMM12 relative to CGM animals, of which 2 genes were upregulated and 31 genes were downregulated (Fig. S2-A). In neonatal sciatic nerve samples, 59 DEGs were regulated between OMM12 and CGM (50 genes were upregulated and 9 were downregulated, Fig. S2-A). The top 7 gene sets that were positively and negatively correlated with OMM12 status were similar to those identified when analyzing GF-derived samples (Fig. S2-B, DRG upper panel, sciatic nerve lower panel). In similarity to comparing GF to CGM status, when comparing OMM12 to CGM status we identified negatively enriched gene sets related to Schwann cell differentiation, myelin sheath formation, neuronal/axonal development and function, cytoskeleton, as extracellular matrix organization and assembly (Fig. S2-C).

#### Findings from young adult animals

**Figure 2.**
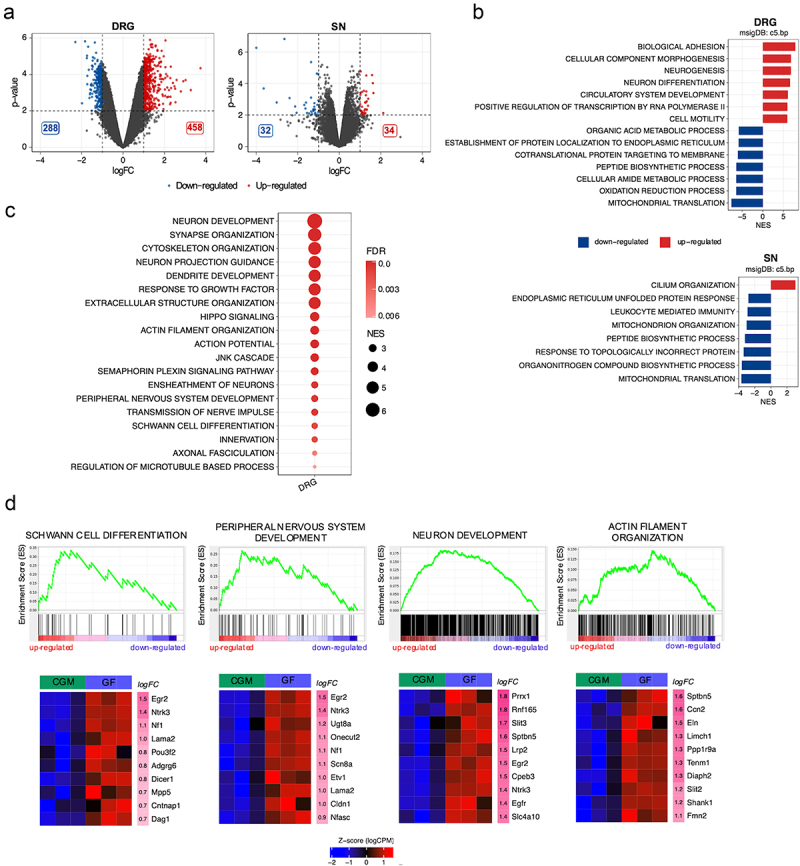
Transcriptomic analysis of young adult GF compared to CGM dorsal root ganglia (DRG) and sciatic nerves (SN). (a) Volcano plots showing differentially expressed genes in young adult DRG and sciatic nerve (GF n = 3; CGM n = 3). Genes with a | log_2_FC | > 1 and with -log_10_(p-value) > 2 are highlighted in red and blue respectively. (b) Top 7 positively correlated (red) and negatively correlated (blue) with the GF status GO biological processes (FDR <0.01) from GSEA of young adult DRG and sciatic nerve. The reported value is the Normalized Enrichment Score (NES). (c) Dot plot showing a list of selected positively enriched GO gene sets related to peripheral nervous system development, maturation and function. Dot size relates to NES value; dot color represents the FDR. (d) GSEA plots of interesting positively enriched GO gene sets found in DRG. Each GSEA plot is associated with a heatmap reporting the expression value as normalized log (CPM) of the top 10 genes in the selected gene set ordered according to logFC in adult CGM and GF. The log_2_FC value is reported in the rightmost column of each heatmap (FDR: false discovery rate; GO: gene ontology).

We detected 746 DEGs regulated in DRG samples when comparing young adult GF with CGM mice (458 upregulated and 288 downregulated, Figure 2a, left panel) and 66 DEGs in sciatic nerve samples when comparing GF with CGM animals (34 upregulated and 32 downregulated, Figure 2a right panel). By performing GSEA, we found that the GO gene sets that exhibited the highest positive correlation with GF status in young adult DRG were related to neuronal and circulatory system development, cell motility, and cell adhesion (Figure 2b, upper panel). The most negatively enriched biological processes were associated with mitochondrial activity, metabolic processes, and protein localization (Figure 2b). In young adult sciatic nerves, GSEA revealed a negative correlation between GF status and mitochondrial activity, the activated immune system, biosynthetic processes, and protein localization, while only one gene set was found to be positively enriched (Figure 2b, lower panel). Intriguingly, the biological processes associated with neurogenesis, axonogenesis, Schwann cell development, myelin formation, and overall development of the PNS were positively enriched in the young adult DRG (Figure 2c). Examples of the most representative GSEA plots and relative heat maps showing the top 10 differentially expressed genes according to logFC are shown in Figure 2d.

By comparing samples from OMM12 and CGM animals, we detected 1935 DEGs in young adult DRG (832 upregulated and 1103 downregulated; Fig. S3-A) and 148 DEGs in the young adult sciatic nerve 53 upregulated and 95 downregulated; Fig. S3-A). GSEA of the DRG samples indicated a positive correlation between OMM12 status and neurogenesis/neuronal differentiation, biological adhesion, and chromatin organization (Fig. S3-B, upper panel). Metabolic processes and mitochondrial activity were negatively correlated with OMM12 status (Fig. S3-B, upper panel). In the sciatic nerve, biological processes associated with chromatin organization and RNA processing were positively enriched, while secretion/exocytosis, immune reaction, cellular adhesion, and extracellular matrix organization were negatively enriched (Fig. S3-B, lower panel).

With a focus on PNS development, GSEA on sciatic nerve data only highlighted the GO “extracellular matrix formation” gene set to be negatively enriched (data not shown), whereas GSEA on DRG data highlighted positively enriched gene sets related to neurogenesis/neuronal development and function, as well as cytoskeleton organization (Fig. S3-C). No gene set associated with myelin sheath and/or Schwann cell differentiation was significantly correlated with OMM12 status, indicating a partial rescue of the molecular profile in OMM12 young adult mice (Fig. S3-C).

### Absence of complex gut microbiota results in smaller diameter and hypermyelinated axons in adult germ-free and gnotobiotic OMM12 mice

In addition to the analysis of molecular changes in the DRG and sciatic nerve, we performed histomorphometrical analysis of another peripheral nerve, the median nerve, to detect possible phenotypic changes.

**Figure 3.**
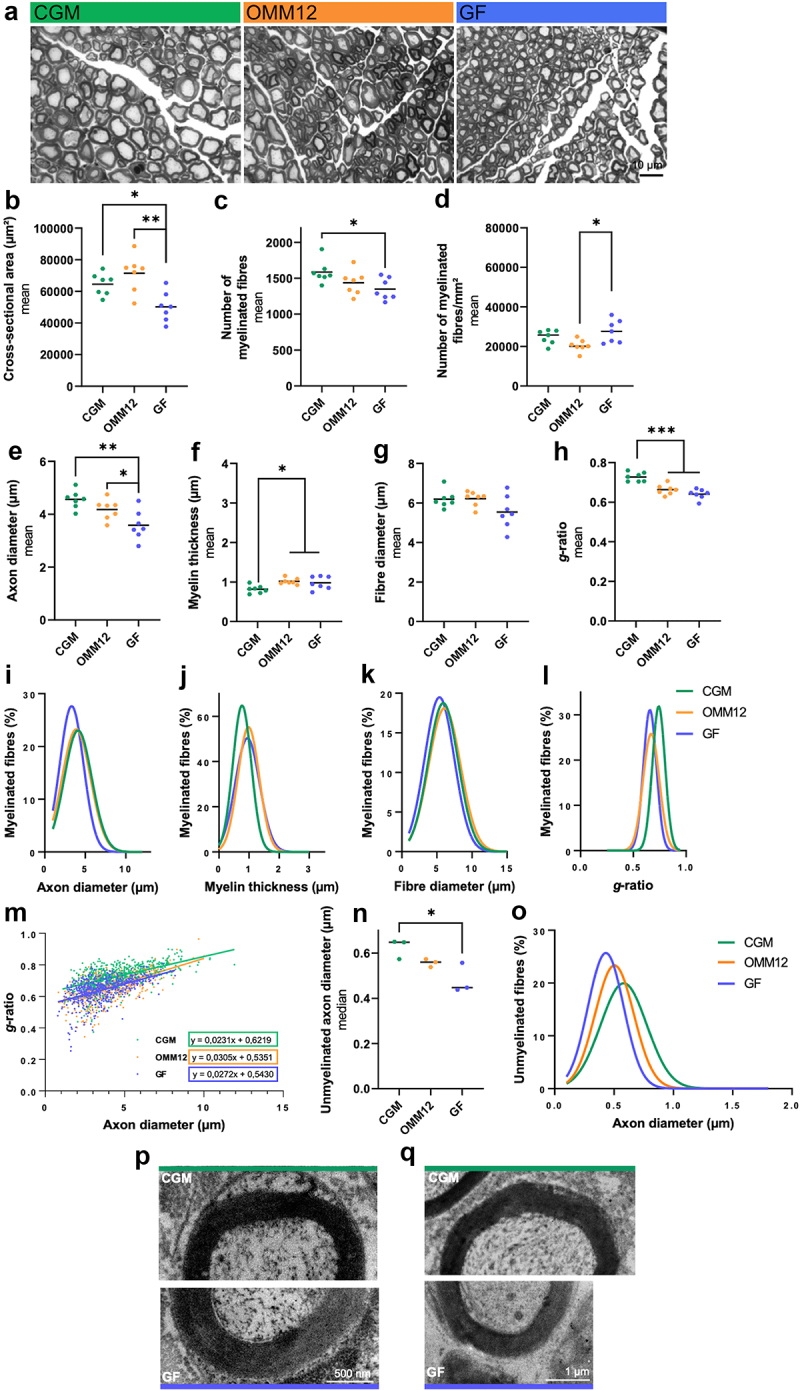
Histomorphometrical and molecular analysis of peripheral nervous tissue. (a) Representative photomicrographs of toluidine blue stained semi-thin cross-sections of the young adult median nerves. (b–d) stereological parameters: cross-sectional area (b), total number of myelinated fibers (c), nerve fiber density (d); (E–H) morphometrical parameters: axon diameter (e), myelin thickness (f), fiber diameter (g), and g-ratio (h). Scatter plots depict the mean of shown individual values. CGM, OMM12, and GF n = 7 animals. Scale bar: 10 µm. Regression curves (Gaussian equation, not given) of percentile distribution for axon diameter (i), myelin thickness (j), fiber diameter (k), and g-ratio (l) and a scatter plot showing the g-ratio of individual myelinated axons as a function of the respective axon diameter (m). Equation of linear regressions are given. CGM, OMM12, and GF n = 560 axons each. (n) Scatter plot of the mean of the axonal diameters of the unmyelinated fibers as detected in median nerves from n = 3 animals per group. (o) Regression curve (Gaussian equation, not given) of percentile distribution for unmyelinated axon diameter. CGM = 474 axons; OMM12 = 393 axons; GF = 405 axons. (p, q) Transmission electron-microscopic comparison of CGM and GF young adult median nerve myelinated axons. (o) Scale bar: 500 nm. (p) Scale bar: 1 µm. Normal distribution was tested using Shapiro–Wilk test. (b–h) Parametric data was subjected to One-Way ANOVA followed by Tukey’s multiple comparisons post-hoc test. (n) Non-parametric data were subjected to Kruskal–Wallis test followed by Dunn’s multiple comparisons post-hoc test. *p ≤0.05, **p ≤0.01, ***p ≤0.001.

No significant differences were detected among median nerves of neonatal CGM, OMM12, and GF mice (Fig. S4), unlike the results obtained in samples from young adult animals. To elucidate the developmental effects, we further performed statistical analysis comparing the data obtained from neonatal and adult mice for each hygiene status (Tab. S1). Stereological parameters were analyzed using toluidine blue-stained semi-thin median nerve cross-sections (Figure 3a). Interestingly, the median nerves of young adult GF mice demonstrated the smallest cross-sectional area (*p* < 0.05) compared to both CGM- and OMM12-derived specimens (Figure 3b). The total number of myelinated nerve fibers did not generally increase compared to neonatal and adult samples (Fig. S4-C, Figure 3c, Tab. S1). The median nerves of the young adult GF animals presented with significantly fewer myelinated fibers than those of the CGM mice (*p* < 0.05) (Figure 3c). However, the resulting nerve fiber density (number of myelinated fibers/mm^2^) of the median nerves harvested from young adult GF mice was not statistically different from that in CGM-derived median nerve specimens, but it was statistically decreased in OMM12-derived samples compared to those of GF mice (*p* < 0.05) (Figure 3d). In parallel, the myelinated fiber density of the median nerve generally decreased when comparing samples from young adult and neonatal animals (Fig. S4-D, Figure 3d). However, the decrease was only significant for the OMM12 status (Tab. S1).

The semi-thin median nerve cross-sections were further analyzed for morphometric parameters of the myelinated fibers. In general, axon diameter, myelin thickness, and fiber diameter significantly increased during development from the neonatal period (Fig. S4-E, F, G) to young adult median nerves in the CGM and OMM12 groups (Figure 3e–g, Tab. S1), whereas the *g*-ratio remained unchanged (Fig. S4-H, Figure 3h, Tab. S1). Mice from the GF status demonstrated a significant increase exclusively in myelin thickness from neonatal to young adults (Figure 3f, Tab. S1). Furthermore, GF-derived samples demonstrated significantly smaller axon diameters than OMM12- (*p* < 0.05) and CGM-derived specimens (*p* < 0.01) (Figure 3e). It is noteworthy that the myelin thickness (Figure 3f) observed in the median nerves of young adult OMM12 and GF mice was significantly increased (*p* < 0.05) compared to samples from CGM mice. The increase in myelin thickness further resulted in the fact that the fiber diameter (Figure 3g) did not differ significantly among the hygiene statuses. Consequently, the *g*-ratio of the median nerves (Figure 3h), reflecting myelin thickness in relation to axon diameter, was decreased in young adult OMM12- (*p* < 0.001) and this was even more pronounced in GF-derived median nerves (*p* < 0.0001) than in CGM-derived specimens.

The morphological differences were additionally displayed as regression curves of the distribution of axon diameter (Fig. S4-I, Figure 3i), myelin thickness (Fig. S4-J, Figure 3j), fiber diameter (Fig. S4-K, Figure 3–k) and *g*-ratio (Fig. S4-L, Figure 3l), which were barely distinguishable for samples from neonatal animal groups. Interestingly, the distributions of axon diameters (Figure 3i) and fiber diameters (Figure 3k) showed a clear left shift for data derived from young adult GF mice, indicating that median nerves comprised an increased number of smaller diameter axons (<5 µm) with a relatively higher myelin volume fraction (hypermyelination) than peripheral nerve fibers in CGM control mice. Hypermyelination is also reflected in the clear right shift in the distribution of myelin thickness (Figure 3j) of both OMM12- and GF-derived data in comparison to CGM-derived data. In young adult OMM12-mice median nerve samples, the slight change toward smaller axonal diameters combined with an increased myelin thickness resulted in a distribution of fiber diameters (Figure 3k) similar to that for specimens derived from young adult CGM mice. A clear left shift of the *g*-ratio data distributions (Figure 3l) derived from OMM12- and GF-specimens compared to young adult CGM-derived data distributions, ultimately underscores that peripheral nerve fibers in young adult GF and OMM12 mice display a hypermyelinated phenotype.

We then plotted scatter plots displaying the *g*-ratio of individual myelinated axons as a function of the respective axon diameter (Fig. S4-M, Figure 3m). For young adults, dots in the lower left area of the plot mainly correspond to GF- and OMM12-derived samples and represent small-diameter axons with relatively thick myelin sheaths (low *g*-ratio) (Figure 3m).

In addition, we analyzed the axonal diameter of unmyelinated fibers and observed significantly smaller unmyelinated nerve fibers in adult GF mice than in CGM mice (Figure 3n). Even in OMM12 derived samples we detected a slight decrease in unmyelinated fiber diameters, resulting in a left shift of the regression curves of distribution for unmyelinated fiber diameter (Figure 3o). Finally, in Figure 3p and q, we have arranged transmission electron-microscopic details of representative myelinated axons in median nerves derived from CGM and GF mice in contrasting juxtaposition.

### Hypermyelination in germ-free mice correlates with dysregulation of Neuregulin 1 (NRG1)/ErbB system

**Figure 4.**
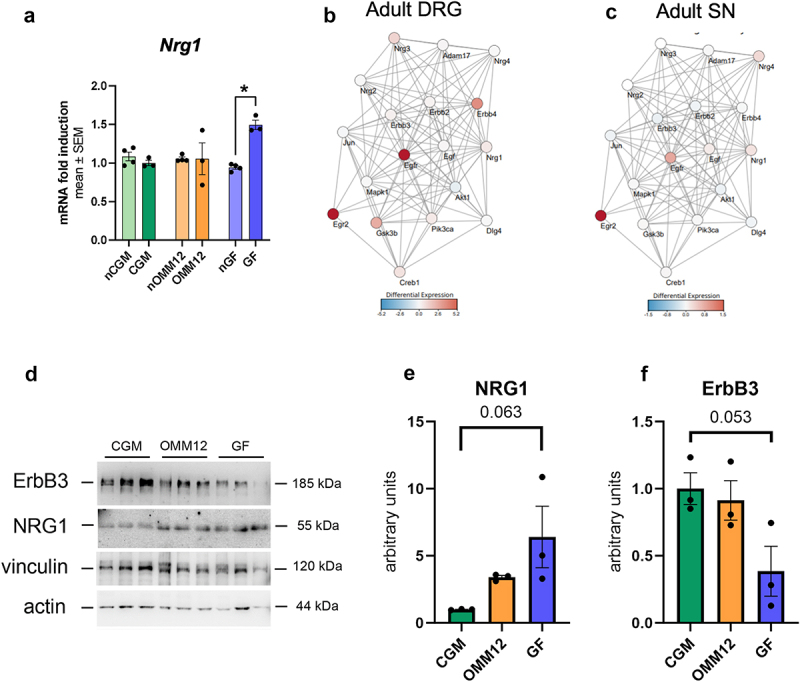
Analysis of NRG1/ErbB system. (a) Expression of *Nrg1* type III mRNA analyzed by qRT-PCR on DRG tissue derived from neonatal and young adult mice. Bar graphs depict the mean ± SEM. nCGM, nOMM12, and nGF n = 4; CGM, OMM12, and GF n = 3 animals. (b-c) Network connecting a selection of genes known to be involved in the NRG1/ErbB pathway. The edges are derived from the STRING database by considering known, predicted and others interactions. The nodes are colored according to the product of the log2(FC) with -log10(p-value) obtained from the comparison between GF and CGM in adult DRG (B) and adult sciatic nerve (c). (d-f) Protein expression analysis of NRG1 type III and its receptor ErbB3 in total protein lysates from sciatic nerves harvested from young adult CGM, OMM12 and GF mice; n = 3 mice. (e-f) Densitometric quantifications of NRG1 type III (e) and ErbB3 (f) normalized to the geometric mean of actin and vinculin densitometric quantifications. Normal distribution was tested using the Shapiro–Wilk test, followed by Kruskal–Wallis test followed by Dunn’s multiple comparisons post-hoc test (a) or by one-way ANOVA with post-hoc Tukey’s test for multiple comparisons (e, f). Data are shown as mean ± SEM; *p ≤0.05.

To explain the previously detected hypermyelination of peripheral axons observed in GF and OMM12 mice, we analyzed the Neuregulin1 (NRG1)/ErbB system, which is known to play a fundamental role in PNS development and nerve repair. In particular, we evaluated with quantitative real-time PCR analysis (qRT-PCR) the expression levels of different isoforms of *Nrg1*, which were not distinguishable in RNA-seq analysis. Soluble *Nrg1* mRNA was barely detectable in all neonatal and adult sciatic nerves, with no differences among the groups (data not shown). In CGM- and OMM12-derived DRG, similar mRNA expression levels were detectable for the transmembrane *Nrg1* isoforms (Figure 4a), while in young adults, compared to neonatal GF-derived DRG, transmembrane *Nrg1* mRNA was significantly upregulated (Figure 4a). We then derived a selection of interactors known to be involved in the NRG1 pathway from STRING and the literature to create an interaction network (Figure 4b–c). Upregulation of some interactors in the NRG1 signaling pathway was observed. In particular, the most upregulated gene in both young adult DRG and sciatic nerve was *Egr2* (Early Growth Response 2, also known as *Krox20*), a transcription factor regulating peripheral myelination (Figure 4b–c).

Finally, protein analysis (Figure 4d) showed an increase in NRG1 type III expression in GF mice (*p* = 0.063, Figure 4e). Interestingly, an opposite expression pattern was observed for the NRG1 receptor ErbB3 (*p* = 0.053, Figure 4f).

### Gut microbiota depletion affects skeletal muscle fiber size

**Figure 5.**
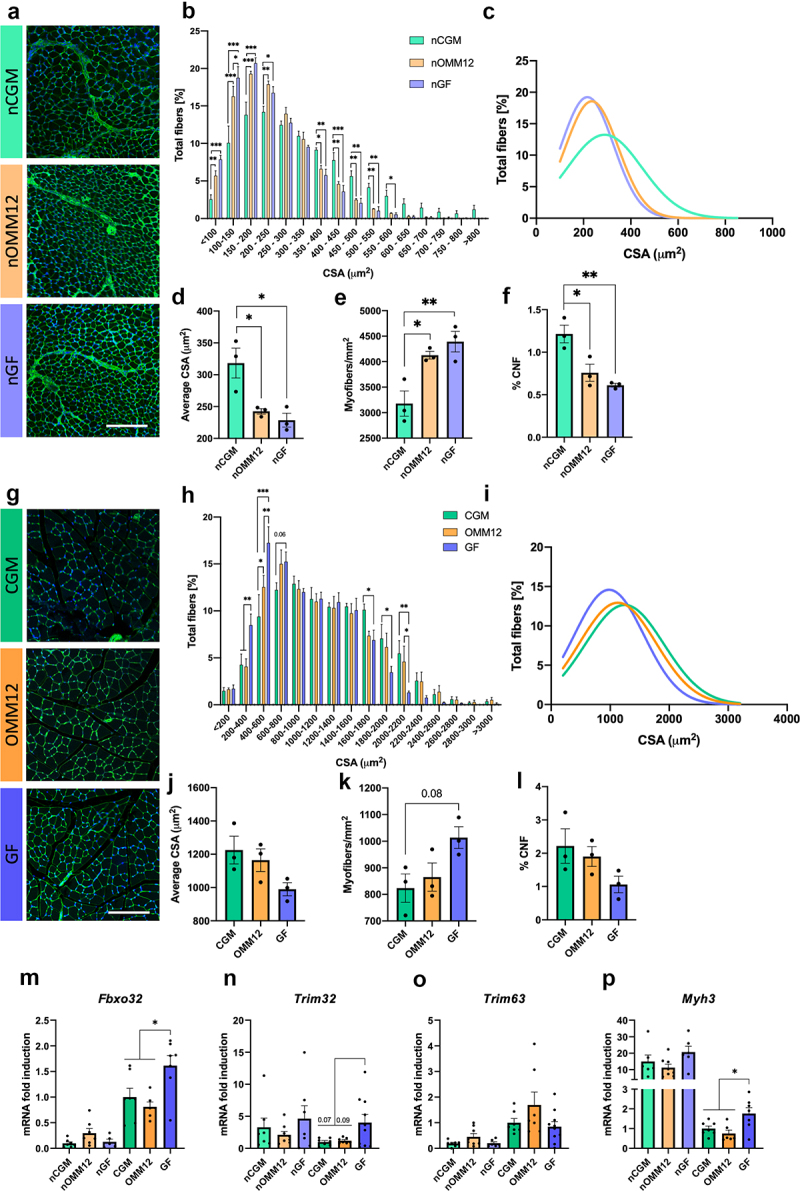
Analysis of muscle atrophy in neonatal and adult muscles. (a) Representative micrographs of neonatal tibialis anterior sections from CGM, OMM12, and GF mice, stained with fluorophore-conjugated wheat germ agglutinin and Hoechst for morphometric analysis. Bar: 150 µm. (b) Cross-sectional area (CSA) distribution among myofibers for the three hygiene status groups of neonatal animals. The comparison between the groups is provided for each class of CSA distribution; two-way ANOVA with post-hoc Tukey’s test for multiple comparisons. (c) Graphical representation of CSA distribution among myofibers. The curves were fitted to data using non-linear regression (Gaussian). (d-f) Average values of CSA, myofiber density and percentage of centrally nucleated fibers (CNF) were evaluated for nCGM, nOMM12 and nGF muscles. Normal distribution was tested using the Shapiro–Wilk test; one-way ANOVA with post-hoc Tukey’s test for multiple comparisons; n = 3 mice, each group. (g) Representative micrographs of young adult tibialis anterior sections from CGM, OMM12, and GF mice. Bar: 150 µm. (h) CSA distribution among myofibers for the three hygiene status groups of young adult animals; two-way ANOVA with post-hoc Tukey’s test for multiple comparisons. (i) Graphical representation of CSA distribution among myofibers. The curves were fitted to data using non-linear regression (Gaussian). (j–l) Average values of CSA, myofiber density and percentage of centrally nucleated fibers (CNF) were evaluated for CGM, OMM12 and GF muscles. Normal distribution was tested using the Shapiro–Wilk test; one-way ANOVA with post-hoc Tukey’s test for multiple comparisons; n = 3 mice/group. (M–P) qRT-PCR analysis for transcripts coding for the atrogenes *Fbxo32* (m) *Trim32* (n) and *Trim63* (o) and the embryonic *Myh3* (p) in mRNA extracts from tibialis anterior of GF, OMM12 and CGM neonatal and young adult mice. Normal distribution was tested using the Shapiro–Wilk test; one-way ANOVA with post-hoc Tukey’s test for multiple comparisons; n = 5–10 mice/group. Bar graphs depict the mean ± SEM; *p ≤0.05, **p ≤0.01, ***p ≤0.001.

To consider the functional target of peripheral nerves and to expand our vision to the neuromuscular system as a whole, we also investigated the impact of microbiota depletion on skeletal muscles. First, a morphometric analysis was performed on neonatal and young adult GF, OMM12, and CGM tibialis anterior muscle cryosections stained with fluorophore-conjugated wheat germ agglutinin and Hoechst (Figure 5a,g). Analysis of myofiber cross-sectional area (CSA) revealed that neonatal muscles from both GF and OMM12 mice contained a remarkably increased percentage of smaller fibers, as well as a reduced percentage of larger fibers, when compared to CGM mice (Figure 5b). This corresponds to a left-shift in the respective distribution curves (Figure 5c). Accordingly, the minimum Feret diameter showed similar results (Fig. S5-A, B). The remarkable shift in myofiber size resulted in a significant reduction in the average cross-sectional area and minimum Feret diameter in GF and OMM12 mice compared with CGM (Figure 5d; Fig. S5-C), with significantly increased myofiber density (Figure 5e). Interestingly, neonatal GF- and OMM12-derived tibialis anterior muscles displayed a reduced percentage of centrally nucleated fibers (Figure 5f). Morphometric analysis conducted in young adult muscles led to similar, although less noticeable, results for GF mice, showing an increased percentage of smaller fibers and decreased percentage of larger ones, in terms of both cross-sectional area and minimum Feret diameter, when compared to CGM muscles (Figure 5h; Fig. S5-D). Conversely, such a shift toward smaller fibers was not observed in samples from young adult OMM12 mice, whose myofiber size distribution appeared to almost overlap with CGM myofibers (Figure 5i, Fig. S5-D). As a result of a weaker effect in young adult muscles, the reduction of the average cross-sectional area and minimum Feret diameter in GF-status muscles, compared to CGM-status muscles, was not statistically significant (Figure 5j, Fig. S5-F), as were the increased myofiber density and the reduced percentage of centrally nucleated fibers (Figure 5k–l).

Myofiber size reduction is predominantly a result of induced atrophic signaling.^24^ To better understand the molecular pathways underlying the changes in morphometry, we performed qRT-PCR for relevant genes associated with the dynamic regulation of muscle mass (Figure 5m–o). Adult GF-derived tibialis anterior muscles displayed increased F-Box Protein 32 (*Fbxo32*, also known as Muscle Atrophy F-box gene or Atrogin-1), and Tripartite Motif Containing 32 *(Trim32)*, although not Tripartite Motif Containing 63 *(Trim63* also known as Muscle RING-finger protein-1, *MuRF-1*), indicating increased protein degradation. Interestingly, neonatal GF and OMM12 muscles, demonstrating even a higher reduction in myofiber cross-sectional area, did not display any variation in the named atrogenes (Figure 5m–o). Of note, only slightly increased atrogene mRNA expression levels were revealed in the analysis of young adult GF soleus muscles, whereas in OMM12-derived specimens the upregulation of some atrogene mRNA levels was more evident in both neonatal and young adult mice (Fig. S6-A, C).

The presence of small caliber myofibers could also represent a sign of regeneration or developmental delay; therefore, we explored the mRNA expression levels of embryonic myosin heavy chain 3 (*Myh3)*. A significant upregulation of *Myh3* was detected in young adults, but not in neonatal GF-derived muscles, when compared with age-matched OMM12- and CGM-derived specimens (Figure 5p, S6-D). This finding may indicate a compensatory adaptive response.

**Figure 6.**
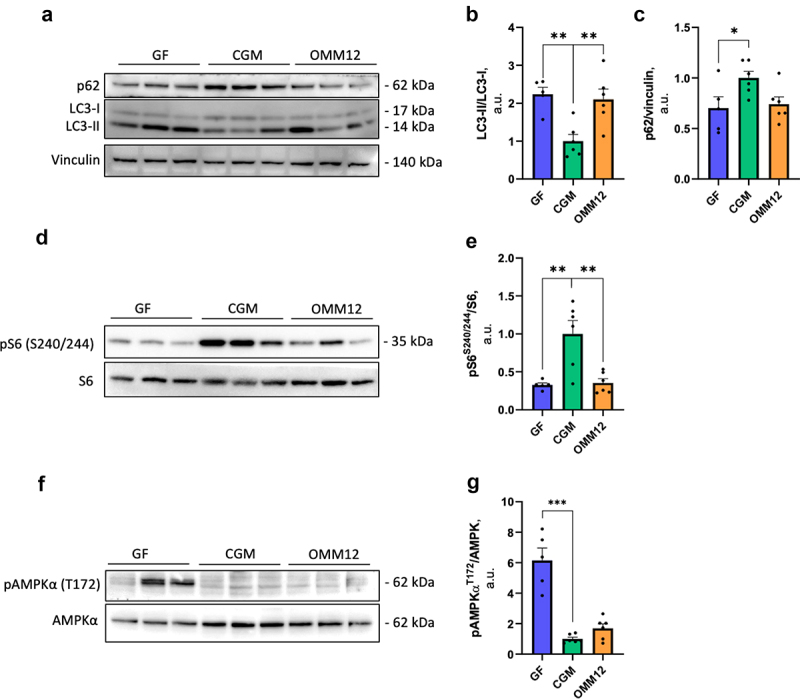
Absence of microbiota induces protein degradation/synthesis and energy imbalance in young adult skeletal muscles. (a) Western blot analysis of the autophagic markers LC3 and p62 in total protein lysates from tibialis anterior muscles harvested from young adult CGM, OMM12 and GF mice. Each image is representative of at least three experimental replicates. (b, c) Densitometric quantifications of LC3-II normalized to LC3-I (b) and of p62 normalized to vinculin as a loading control (c), as determined by three independent experiments. Normal distribution was tested using the Shapiro–Wilk test; one-way ANOVA with post-hoc Tukey’s test for multiple comparisons; n = 6 mice, for CGM and OMM12; n = 5 mice for GF. (d) Western blot analysis of S6 phosphorylation levels in total protein lysates from tibialis anterior muscles harvested from young adult CGM, OMM12 and GF mice. Each image is representative of at least three experimental replicates. (e) Densitometric analysis of phosphorylated S6 (p-S6) normalized to total S6 protein expression, as determined by at least three independent experiments. Normal distribution was tested using the Shapiro–Wilk test; one-way ANOVA with post-hoc Tukey’s test for multiple comparisons; n = 6 mice, for CGM and OMM12; n = 5 mice for GF. (f) Western blot analysis of AMPKα phosphorylation levels in total protein lysates from tibialis anterior muscles harvested from young adult CGM, OMM12 and GF mice. Each image is representative of at least two experimental replicates. (e) Densitometric analysis of phosphorylated AMPKα (pAMPKα) normalized to total AMPKα protein expression, as determined by at least two independent experiments. Normal distribution was tested using the Shapiro–Wilk test; one-way ANOVA with post-hoc Tukey’s test for multiple comparisons; n = 6 mice, for CGM and OMM12; n = 5 mice for GF. Data are shown as mean ± SEM; *p ≤0.05, **p ≤0.01, ***p ≤0.001; a.U., arbitrary units.

To further investigate the presence of an imbalance in protein synthesis and muscle mass remodeling in young adult muscles, the levels of some specific autophagy markers, including microtubule-associated protein light chain 3 1A/1B-light chain 3 (LC3), a gold standard autophagosome marker (when lipidated in its LC3-II form), and ubiquitin-binding protein p62 (p62), an eat-me-signal whose levels inversely correlated with activated autophagy,^25^ were analyzed (Figure 6). While only GF mice showed markedly reduced p62 levels, both GF and OMM12 tibialis anterior muscles displayed significantly higher levels of LC3-II than CGM mice (Figure 6a–c), suggesting that autophagy is modulated in skeletal muscles by alterations in the microbiota. In parallel to an enhancement of degradative pathways, protein synthesis is reduced, since phosphorylation of the ribosomal protein S6, a common readout for mammalian target of rapamycin complex 1 (mTORC1) activation,^26^ appeared strongly reduced in both GF and OMM12 muscles when compared to CGM (Figure 6d–e). Interestingly, GF tibialis anterior muscles, but not OMM12 muscles, displayed increased levels of phosphorylated AMP-activated protein kinase (AMPK) (Figure 6f–g), a sensor for energy imbalance.^27^

### Germ-free mice display signs of neuromuscular junction remodelling

**Figure 7.**
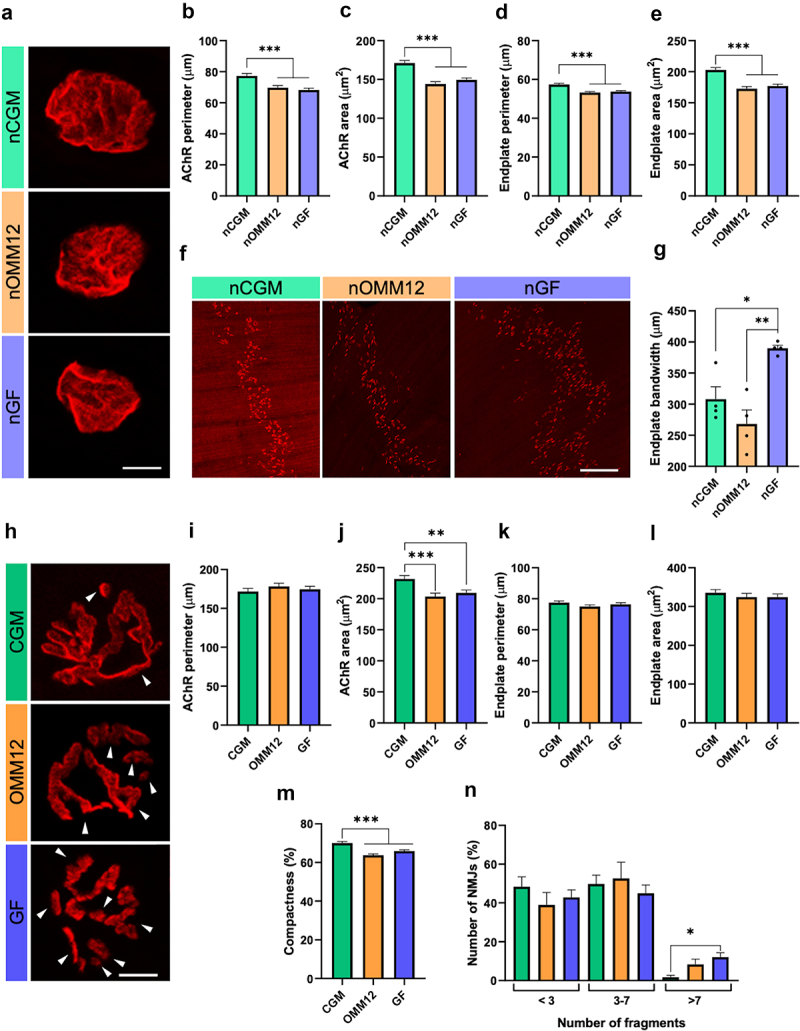
NMJ morphometric analysis in neonatal and young adult mice. (a) Representative images of α-bungarotoxin-stained NMJs in whole-mount preparation of CGM, OMM12 and GF neonatal diaphragms, used for morphometric analysis with the aNMJ-morph platform. Scale bar, 10 μm. (b-e). Quantitative analysis of postsynaptic variables from diaphragm muscles of CGM, OMM12 and GF neonatal mice showing AChR perimeter (b), AChR area (c), endplate perimeter (d), and endplate area (e). Normal distribution was tested using the Shapiro–Wilk test; Kruskal–Wallis test with post-hoc Dunn’s test for multiple comparisons; NMJs n = 213 for CGM; n = 221 for OMM12; n = 282 for GF. (f) Representative images of α-bungarotoxin-stained whole-mount preparation of nCGM, nOMM12 and nGF diaphragms, used for endplate bandwidth measurements (g). Scale bar, 200 μm. Normal distribution was tested using the Shapiro–Wilk test; one-way ANOVA with post-hoc Tukey’s test for multiple comparisons; n = 4 mice, each group. (h) Representative images of α-bungarotoxin-stained NMJs in adult diaphragms. White arrowheads indicate isolated clusters, counted as fragments. Scale bar, 10 μm. (i-m) Quantitative analysis of postsynaptic variables from diaphragm muscles of CGM, OMM12 and GF adult mice showing AChR perimeter (i), AChR area (j), endplate perimeter (k), endplate area (l), and compactness (m). Normal distribution was tested using the Shapiro–Wilk test; Kruskal–Wallis test with post-hoc Dunn’s test for multiple comparisons; NMJs n = 144 for CGM; n = 168 for OMM12; n = 171 for GF. (n) NMJs from young adult CGM, OMM12 and GF mice were classified into three categories, according to the number of isolated fragments of AChR clusters observed. The number of NMJs belonging to each group was calculated as a percentage of the total NMJs number for each hygiene status group. Normal distribution was tested using the Shapiro–Wilk test; one-way ANOVA with post-hoc Tukey’s test for multiple comparisons; n = 4 mice, each group. Data are shown as mean ± SEM; *p ≤0.05, **p ≤0.01, ***p ≤0.001.

The neuromuscular junction (NMJ) is a functional system that results from the contact of motor axons with skeletal muscle fibers. To investigate whether NMJs revealed any other relevant defect when comparing the three mouse models, NMJ morphometry was analyzed in whole-mount diaphragms stained with labeled α-bungarotoxin and evaluated using the macro NMJ-morph.^28^ Five postsynaptic variables were measured: acetylcholine receptor (AChR) perimeter and area, endplate perimeter and area, and NMJ compactness. The analysis performed in neonatal diaphragms revealed a remarkable reduction in AChR perimeter and area as well as in endplate perimeter and area of both GF and OMM12 NMJs (Figure 7a–e), indicating that these NMJs are smaller and less developed than neonatal CGM ones. However, no major differences were detected in terms of compactness, number of fragments, or fragmentation index (Fig. S7-A-C). Moreover, analysis of the AChR cluster bandwidth demonstrated a wider distribution of NMJs, indicating a more immature or altered innervation of NMJs in GF mice than in OMM12 and CGM mice (Figure 7f–g). Morphometric analysis of diaphragms derived from young adult mice (Figure 7h–n), revealed a significant reduction in AChR area and compactness (Figure 7j–m), reflecting the presence of a reduced distribution of AChR clusters at the endplate in both OMM12 and GF compared to CGM young adult mice. Furthermore, an increased percentage of NMJs displaying a higher number of fragments was detected in GF-derived young adult diaphragms (Figure 7n), although it did not significantly affect the mean number of fragments or fragmentation index (Fig. S7-D, E). Of note, NMJs with more than 9 were detectable in GF- and OMM12-derived young adult diaphragms, but not in CGM (Fig. S7-F).

**Figure 8.**
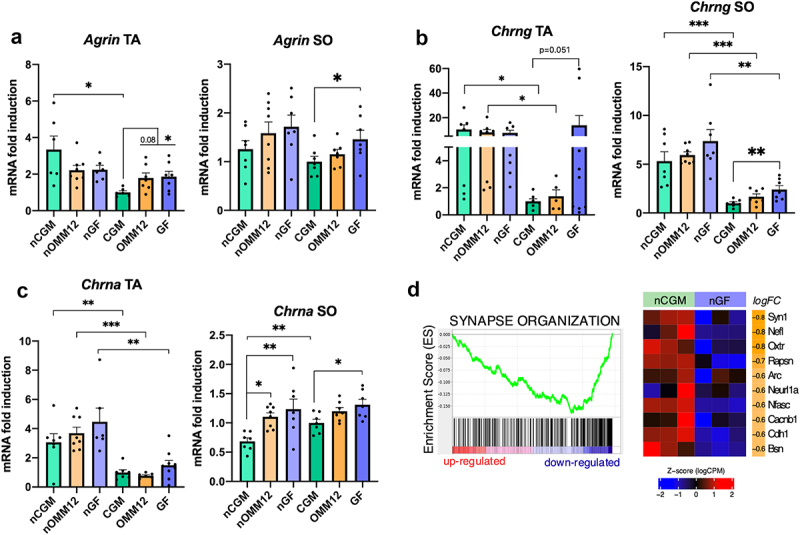
Expression of NMJ-related genes. RT-qPCR analysis for transcripts coding for the synaptic genes: (a) *Agrin*, (b) *Chrng* and (c) *Chrna* in tibialis anterior and soleus muscles; one-way ANOVA with post-hoc Dunnett’s test for multiple comparisons within the same age; unpaired two-tailed Student’s t test newborn vs adult, same group; normal distribution tested using the Shapiro–Wilk test; non-parametric data were subjected to Mann–Whitney test (GF, *Chrng*). Bar graphs depict the mean ± SEM; *p ≤0.05, **p ≤0.01, ***p ≤0.001. (d) On the left, GSEA plot of the GO biological process “SYNAPSE ORGANIZATION” obtained performing GSEA on the comparison between neonatal GF and CGM sciatic nerves. On the right, heatmap reporting the expression value as normalized log (CPM) of the top 10 genes in the selected gene set ordered according to logFC in neonatal CGM and GF. The logFC value is reported in the rightmost column of the heatmap (Ta = tibialis anterior; SO=soleus muscle).

We additionally investigated the presence of alterations in NMJ-related gene expression using qRT-PCR in both tibialis anterior and soleus muscle specimens. Interestingly, mRNA levels of *Agrin*, encoding an extracellular-secreted proteoglycan mainly involved in orchestrating AChR clusters at the membrane,^29^ were upregulated in young adult GF mice compared to CGM-derived specimens of both muscles (Figure 8a). Likewise, the expression of the *Chrng* gene, which encodes the embryonic γ subunit of AChR (Figure 8b), and the *Chrna* gene, which encodes the α subunit, whose expression is typically regulated by electrical activity,^30^ was upregulated (Figure 8c). Interestingly, samples harvested from young adult OMM12 mice did not show significant upregulation of the same genes compared to CGM samples (Figure 8a–c).

Consistently, the previously described GSEA of sciatic nerve identified negatively enriched sets associated with synapse assembly and synapse vesicle functions, as well as regulation of neurotransmitter level, transport, and secretion (Figure 8d), highlighting an impairment in the correct development of nerve-muscle crosstalk upon microbiota depletion.

## Discussion

The gut microbiota is known to benefit host physiology, influencing the development of various organs, including the brain and enteric nervous system. However, whether gut microbiota contributes to somatic peripheral nervous system (PNS) development remains unclear. In the present study, we show for the first time that the absence of a complete gut microbiota during prenatal and postnatal periods impairs the development of peripheral nerves and their motor targets.

To reveal differences in gene expression among GF, OMM12, and CGM mice, we used high-throughput RNA sequencing to compare the entire transcriptome of the dorsal root ganglia (DRG) and sciatic nerves. At the gene expression level, peripheral glial and sensory neuron development was impaired or delayed in GF status as compared to CGM status. In neonatal GF animals, the gene sets related to PNS development and myelination were negatively enriched, and the same was positively enriched in young adult animals. This suggests either a delay in PNS development or a rescue attempt to be related to the GF status initiated in the young adult condition and already reached in neonatal CGM tissue. The same can be concluded for OMM12 mice, in which PNS glial development appears to be perturbed in neonates and rescued in young adults compared to GF mice.

Our results are in accordance with studies on the enteric nervous system (ENS), a branch of the PNS that provides intrinsic innervation of the bowel.^16,31^ Early exposure to intestinal bacteria is essential for ENS postnatal development. GF mice show reduced nerve fiber density and fewer neuronal cells in their myenteric plexus compared to specific pathogen-free mice.^32^ Gut microbiota also influences the maturation of intestinal neural networks and the functional activity of the ENS and is therefore essential for normal gut – brain signaling.^18,33^ Interestingly, colonization of GF mice with conventional microbiota normalized the neuroanatomy of the ENS and gut physiology by inducing maturation of neuronal precursors in the myenteric plexus.^18^

Our morpho-quantitative analysis revealed significantly reduced myelinated and unmyelinated axonal diameters in young adult GF mice, indicating abnormal peripheral nerve fiber development. However, as indicated by the reduced *g*-ratio, the myelin sheaths of myelinated fibers are relatively too thick for the respective axonal diameters. This was also detected in the OMM12-status, where axonal diameters of myelinated and unmyelinated fibers were only slightly reduced, but myelin thickness increased in comparison to the CGM status. Taken together, these results suggest that the absence of a complete gut microbiota leads to hypermyelination of peripheral axons whose diameters are reduced (probably with a compensatory role), and that the 12 bacterial strains in the gut of OMM12 animals are not sufficient to rescue the observed hypermyelination phenotype of peripheral nerves in young adult mice. Our results are in accordance with those of previous studies, demonstrating that removing gut microbiota may contribute to hypermyelination in the CNS. Hoban *et al*. demonstrated that GF mice exhibit hypermyelination of fibers of the prefrontal cortex in comparison to conventionally raised animals.^34^ In addition, callosal myelination has been shown to be altered in a model of Huntington’s disease: comparison of specific pathogen-free and GF mice showed similar *g*-ratios, whereas BACHD mice (a transgenic model of Huntington’s disease) raised in GF status showed lower *g-*ratios compared to BACHD mice raised in specific pathogen-free status.^35^ Finally, the dysbiosis induced by neonatal treatment with antibiotics has been shown to impair myelination in the adult brain; myelin-related genes as well as immunoreactivity for myelin basic protein are upregulated in the prefrontal cortex region of antibiotics-treated mice compared to sham-treated controls, suggesting the presence of more myelin in the prefrontal cortex region upon antibiotics treatment.^36^ Taken together, these data indicate hypermyelination of central fibers as a consequence of the absence of a complete gut microbiota, while our data, for the first time, demonstrate the same for somatic peripheral nerve fibers.

To partly explain the hypermyelination in young adult nerves, we analyzed the expression of different isoforms of the EGF-like growth factor Neuregulin1 (*Nrg1*). Different NRG1 isoforms play distinctive roles in Schwann cell activity and myelination. Transmembrane NRG1 type III expressed by axons is an essential neuronal signal for Schwann cell myelination,^37,38^ whereas soluble NRG1 type I/II is mainly involved in Schwann cell reprogramming after nerve injury.^39^ Intriguingly, Nrg1 Type III was upregulated in young adult GF mice, suggesting altered NRG1 signaling during myelin maturation in the absence of gut microbiota. These results align with the literature, showing a role of NRG1 Type III in rescuing myelination defects in *Nrg1* Type III-/- neurons,^37^ and in restoring myelination defects in a model of congenital hypomyelinating neuropathy.^40^ STRING analysis performed on RNA-seq data revealed upregulated NRG1 interactors in GF mice, including NRG1 receptors (*ErbB4* and *Egfr*) and the transcription factor *Egr2* (also known as Krox-20), a master regulator of myelination activated by NRG1 Type III.^41–43^ Despite the upregulation of both *Nrg1* type III and *Egr2*, myelin-related transcripts were not upregulated (data not shown). This suggests that the regulation of these genes occurred at an earlier stage of development, and they were subsequently deactivated to achieve a stable state of myelin maintenance.^41^ Moreover, unmyelinated fibers in *Nrg1* Type III conditional knockout mice have larger diameters,^44^ suggesting that *Nrg1* type III expression is inversely correlated with unmyelinated fiber size. Accordingly, GF mice, where *Nrg1* type III expression is higher, have smaller unmyelinated fibers than CGM mice. Taken together, these results suggest that gut microbiota depletion affects the *Nrg1* type III pathway, thus impairing the correct development of both myelinated and unmyelinated fibers.

Skeletal muscles, target effectors of peripheral nerve fibers, are also affected by gut microbiota composition. The onset of an atrophic phenotype in young adult GF mice was in accordance with previous studies,^45^ which also show a reduced grip strength and swimming endurance capacity in young adult GF mice when compared with age-matched conventionally raised mice.^45–47^ However, the results obtained from neonatal mice in our study show, for the first time, a clear reduction in muscle fiber size in GF mice and that the colonization of the gut with OMM12 is not sufficient to rescue such reduction at this developmental stage. At later stages, gut colonization with OMM12 accounted for the presence of morphometric muscle features much more similar to those in specimens harvested from CGM mice than from GF mice.

The reduction in muscle fiber size in GF animals appeared to be sustained by activated atrophy signals, as witnessed by *Fbxo32* and *Trim32* upregulation in young adults, but not in neonates, indicating slower development in the latter case. Embryonic *Myh3* was significantly upregulated in young adults but not in neonates. The upregulation of developmental myosins concurrently with the onset of atrophy induction was reported in sarcopenic muscles as a result of a failure of compensatory mechanisms to counteract muscle mass loss.^48^ Muscle atrophy induced by microbiota depletion appears to be a result of an imbalance between protein degradation and protein synthesis, which was also verified by the induction of the autophagy process and by signals revealing limited protein synthesis and energy imbalance, such as the reduced phosphorylation of ribosomal protein S6 and the increased phosphorylation of AMPK, respectively.^49^

Our analyses demonstrated that both peripheral nerves and muscles are affected by the gut microbiota status, morphology, and composition. Therefore, we investigated whether NMJs revealed other relevant defects when comparing the three hygiene statuses. Morphometric parameters in neonatal GF and OMM12 NMJs are consistent with an immature phenotype.^50^ While not relevant at the neonatal stage, when postsynaptic endplates still appear mostly as a unique plaque with few major perforations,^51^ the high fragmentation detected in young adult GF diaphragms is described as an admitted sign of NMJ degeneration, typically observed upon muscle denervation, physiological aging and atrophy.^52,53^ Recently, however, high NMJ fragmentation has also been discussed as a readout for myofiber regeneration.^54^ In this context, more fragmented NMJ and upregulated *Chrng* and *Chrna* are consistent with both a denervation-like phenotype and a regenerative phenotype, while the increase in *Myh3* expression indicates a regeneration-related feature in the GF status. However, a third explanation could be considered for detecting such a fragmented phenotype in adult GF NMJs. Previous studies from Lee *et al*. demonstrated that overexpression of *Nrg1* type III (but not *Nrg1* type I/II) in motor neurons and DRG leads to hypermyelination and NMJ fragmentation, occurring without evident signs of denervation, and consistent with a delay in the developmental stage of synapse elimination.^55^ This appears to be in line with the delayed development of the neuromuscular system we observed in GF mice. In accordance with these data, sciatic nerve transcriptomic analysis revealed downregulated pathways related to synaptic vesicle activities and regulation of neurotransmitter levels, transport, and secretion in neonatal GF mice, suggesting an impairment in peripheral nerve-muscle crosstalk.

Acetate, propionate, and butyrate are the main short-chain fatty acids produced by bacterial fermentation of dietary fibers and resistant starch, and growing evidence supports the idea that they exert crucial physiological effects on several organs, including the central and enteric nervous system.^16,56^ Recently, the impact of neonatal antibiotics-induced dysbiosis on the gut-brain axis, including myelination, has been studied, showing that neonatal antibiotics administration led to increased myelination in the prefrontal cortex in adulthood. Interestingly, these alterations were restored by butyrate administration,^36^ suggesting a critical role of butyrate in the process of CNS myelination. In a recent study, the metabolic potential of individual members of the OMM12 community was characterized *in vitro* and the main producers of short-chain fatty acids were identified.^57^ In OMM12-status, butyric acid is only produced by *F. plautii* YL31 and *C. innocuum* I46,^57^ whereas in the complex gut microbiota status, several *genera* (*Ruminococcus, Clostridium, Eubacterium, and Coprococcus*) are common butyrate-producing bacteria,^58^ suggesting a consequent higher metabolite production. Moreover, butyrate-producing bacteria have also been positively associated to muscle health, strength and function, even in sarcopenic conditions,^59,60^ highlighting the important role of these bacteria in the correct development and functioning of the whole neuromuscular system. However, the possibility that other metabolites such as polyamines, bile acids, amino acids, carbohydrates are responsible for the observed phenotypes cannot be fully excluded. Today we cannot explain how the differences in bacterial composition and metabolite profiles between OMM12 and CGM account for the phenotypic (hypermyelination) and transcriptomic differences demonstrated. Our KEGG annotation analysis, however, points toward metabolic differences relevant for the nervous system and future work is needed to define the precise relationship between microbial species, gut-derived metabolites, myelinating cells in the somatic PNS and nerve/muscle crosstalk.

Recently, one new minimal bacterial community OMM19.1 was developed.^61^ This community was generated by amending the OMM12 community with nine additional bacterial species to add specific functions to the consortium that render complex microbiota such as addition of phylogenetic diversity, secondary bile acid production, and equol production. Addition of these functions increased the similarity between the OMM19.1 model and complex microbiota colonized mice in terms of body composition, immune cells in the intestine and associated lymphoid tissues. However, even though the phenotypic similarity of OMM19.1 to complex colonized mice was higher than the one of OMM12, this model, for several physiological aspects, still represents an intermediate state between OMM12 mice and the ones colonized by a complex natural microbiota. In this study we show that not microbial signals in general, but rather specific microbial factors are responsible for adequate PNS structure development. These could indeed be the missing function in OMM12 such as secondary bile acid production, but also other microbial signals.

In conclusion, we present here, for the first time to our knowledge, evidence for a direct impact of gut microbiota on somatic PNS development, and we propose the existence of a novel ‘GM-PNS-axis’. The identification of this axis opens new horizons for further investigations that will lead to a better understanding of the role of microbiota in PNS physiological/pathological and regenerative conditions. Recent studies have demonstrated the crucial role of the gut microbiota in nerve regeneration and functional recovery after traumatic nerve injury.^62,63^ Our and other new data on the gut microbiota-PNS axis pave the way for possible future development of innovative microbiota-based therapies to improve the prognosis and progression of a growing list of PNS-related pathological conditions and diseases.

## Materials and methods

### Mice

Neonatal (P8–14) and young adult (63–67 days old) germ-free (GF), gnotobiotic (colonized with Oligo-Mouse-Microbiota 12, OMM12), and specific pathogen-free C57BL6/JZtm (harboring complex gut microbiota/CGM) mice were obtained from the Central Animal Facility (Hannover Medical School, MHH, Hannover, Germany). Microbiota-colonized mice (CGM and OMM12) were generated by colonizing GF C57BL6/JZtm mice with their respective microbiota. Mice born with this microbiota or their offspring were used in this study. The colonization procedure was repeated every 10 generations to avoid the formation of substrains. Breeding and maintenance of GF and gnotobiotic OMM12 mice were performed in plastic film isolators (Metall+Plastik GmbH, Radolfzell-Stahringen, Germany) located in a room with a controlled environment (20–22°C, 50–55% humidity) and 12-hour light/dark cycles. GF and gnotobiotic OMM12 mice received pelleted 50 kGy gamma-irradiated feed (V1124–927, Ssniff Spezialdiäten GmbH, Germany) containing 14% fat, 27% proteins, 59% carbohydrates, and autoclaved water ad libitum. Specific pathogen-free mice were housed in individually ventilated cages (XJ Edge, Allentown) in a room with a controlled environment (20–22°C, 50–55% humidity) and 12 h light/dark cycles. Specific pathogen-free mice received pelleted 50 kGy gamma-irradiated feed (1314 breeding diet for mice and rats, Altromin) containing 12% fat, 26% proteins, 62% carbohydrates, and chlorinated water, ad libitum. The GF and gnotobiotic colonies are extensively and rigorously tested to confirm their microbiological status. The Central Animal Facility (MHH, Hannover, Germany) protocols for screening of gnotobiotic colonies include a variety of methods including microbiological and molecular biology methods. Fecal pellets, environmental samples including used food, bedding and swabs from isolators are analyzed once a month for bacterial and fungal contaminations by direct cultivation in thioglycolate and Sabouraud dextrose broth under aerobic conditions at 37°C, 29°C, and room temperature for at least 10 days. Thioglycolate broth is a multipurpose, enriched, differential medium used for determination of microorganisms’ oxygen requirements. As the oxygen concentration decreases from the top to the bottom of the glass tube it supports the growth of aerobes, anaerobes, microaerophilic, and fastidious microorganisms. Sabouraud dextrose broth is a complex medium for cultivation of yeasts, molds and dermatophytes. Additionally, mold traps are continuously kept in isolators to detect the presence of molds or yeasts within the isolator. Every 6 months live animals (retired breeders) are sacrificed for the purpose of monitoring the presence of specific mouse pathogens based on the FELASA recommendation list^64^ and confirming their gnotobiotic status. In this context, cultural analysis of cecal and colonic intestinal content, as well as native preparations of cecum smears using phase contrast microscopy are performed. Additionally, critical evaluation of the cecum size is also one of the screening criteria, as the shrinked cecum in germ-free animals can point to the microbial contamination. Breeding colonies of animals colonized with defined microbial species are additionally screened by 16S rRNA-based microbe-specific qPCR and next-generation sequencing to confirm the presence of intentionally introduced microorganisms and to exclude the presence of unknown/unwanted contaminants.^65^ Routine microbiological monitoring according to the FELASA recommendations^64^ and recommendations for maintaining gnotobiotic colonies^66^ did not reveal any evidence of infection with common murine pathogens in specific pathogen-free animals or contaminants in GF and gnotobiotic animals.

This study included tissue samples from 42 adult and 41 neonatal animals. Each animal served as a single experimental unit during our study; the animals for the analysis were collected sequentially over a period of several months from stable breeding colonies (CGM, OMM12 and GF) depending on their availability. The animals were sacrificed by decapitation (adult ones after inducing anesthesia in CO_2_ atmosphere). All procedures were performed in accordance with the German Animal Welfare Legislation and the principles of the Basel Declaration and recommendations of Directive 2010/63/EU. All procedures were approved by the local Institutional Animal Care and Research Advisory Committee and registered with the Animal Care Committee of Lower-Saxony, Germany. Breeding and animal husbandry was registered under the number 42,500/1 H according to §11 of the German protection of animal act (TierSchG). Animal sacrifice for scientific purposes was announced to the authorities under the numbers §4 2021–289 (neonatal mice) and §4 2017–171 (adult mice).

### DNA isolation, shallow shotgun metagenomics and sequencing bioinformatic analysis

For shotgun metagenomic analysis of the gut microbiota from young adult CGM and OMM12 mice fecal pellets were collected from respective stable breeding colonies. Fecal pellets from *n* = 4 age-matched CGM and OMM12 mice were then processed to DNA isolation and shotgun metagenomics for taxonomical profiling of the gut microbiota. DNA isolation from fecal samples was performed using the PSP® Spin Stool DNA Basic Kit (Invitek Molecular GmbH, Germany) according to the manufacturer’s instructions. Eluted DNA was stored at ‑80°C until shipment to the company “Novogene” that performed shallow shot metagenomics and bioinformatic analysis.

DNA samples were first randomly fragmented to a size of 350 bp using a Covaris ultrasonicator. This was followed by end repair, dA tailing, adaptor ligation, and purification/PCR amplification. The generated libraries were quantified using Qubit Fluorometer, then diluted to 2 ng/μl. The diluted libraries were further assessed for size distribution by Agilent 2100 Bioanalyzer and quantified again using real-time PCR (final concentration >3 nM was required). Library QC was performed, and qualified libraries were pooled based on library concentration and desired read depth (2 G raw data per sample). Pooled libraries were sequenced on Illumina NovaSeq 6000 instrument with paired end 150 bp (PE150) strategy.

Raw data from the Illumina sequencing platform were preprocessed using Readfq (https://github.com/cjfields/readfq.) to obtain clean data for analysis using following steps: a) Removing reads with low-quality bases (default quality threshold is ≤38) that exceed a certain proportion (default length is 40 bp); b) Removing reads with N bases reaching a certain proportion (default length is 10 bp); c) Removing reads whose overlaps with adapters exceed a certain threshold (default length is 15 bp). Subsequently, clean data were BLASTed to the host database to filter out reads coming from the host. Bowtie2 software (http://bowtie-bio.sourceforge.net/bowtie2/index.shtml) is used by default, with the following parameter settings: –end-to-end, –sensitive, -I 200, and -X 400.^67–69^ Metagenomes were assembled using MEGAHIT software with assembly parameter settings: –presets meta-large (–end-to-end, –sensitive, -I 200, -X 400),^68,70^ and Scaftigs without N is obtained by breaking the resulted Scaffolds from the N junction.^71,72^ Species annotation was performed using DIAMOND software (https://github.com/bbuchfink/diamond/.)^73^ by alignment of Unigenes sequences with those of bacteria, fungi, archaea, and viruses extracted from NCBI’s NR database (https://www.ncbi.nlm.nih.gov/), with parameter settings: blastp, e 1e-5.^68^ From the alignment results of each sequence, the one with evalue ≤ min. evalue * 10 is selected. Since each sequence may have multiple alignment results, LCA algorithm (applied to systematic taxonomy of MEGAN software (https://en.wikipedia.org/wiki/Lowest_common_ancestor) is adopted to determine the species annotation information of the sequence.^74^ According to the abundance table of each taxonomic level, principal coordinates analysis and bar plot analyses for abundant taxa were performed. Annotations for common functional database KEGG were performed with DIAMOND software ((https://github.com/bbuchfink/diamond/.) to align Unigenes with those in the functional KEGG database (http://www.kegg.jp/kegg/).^75^ From the alignment results of each sequence, the Best Blast Hit results are selected for subsequent analysis.^76–79^ The gene number table of each sample at each taxonomy level is derived from the result of functional annotation and gene abundance table. The number of genes with a certain function in a sample is equal to the number of genes whose abundance is non-zero among the genes annotated with this function.

### Cross-validation of bacterial colonization of the OMM12 mice

The PE150 sequencing reads derived from the OMM12 mice fecal pellets fraction generated by the shallow shot metagenomics analysis were processed in parallel using the whole metagenome sequencing pipeline “Wochenende” version: 2.0.0 - Nov 2021 (https://github.com/MHH-RCUG/nf_wochenende/wiki). This pipeline implements both quality analysis as well as a module for aligning the short reads against a reference database.^80^ A customized in-house reference database curated by the Central Animal Facility at the MHH was used, which includes one mice genome as a reference sequence representing the host and one completely sequenced, quality-controlled genome per bacterial (*n* = 12) species of the Oligo-MM genomes (overview in Table 1 of)^81^. The Reference database is available on request, containing the mouse genome mm10 plus OMM bacterial species. The analysis was performed using default parameters for the “Wochenende” pipeline, incorporating the minimap2short aligner, with a maximum of 2 mismatches allowed, filtering reads from multi-mapping alignments at quality 30 (MQ30) which contain little specific phylogenetic signal. Normalization was realized with Wochenende submodules. To validate the uniform read distribution across a reference genome of a distinct species the so-called raspir module was applied.^82^ A second metagenome analysis pipeline with default settings was applied to determine hypotheses regarding the origin of the sequencing reads which might occur as background from the mice nutrition. KrakenUniq,^83^ Version 1.0.4, has been well described already elsewhere. The standard reference database, available from https://benlangmead.github.io/aws-indexes/k2 was incorporated for the analysis.

### RNA extraction and RNA-seq library preparation and analysis

For RNA-seq analysis of sciatic nerves, for each sample, we pooled the two sciatic nerves belonging to the same animal, while for RNA-seq analysis on DRG, we pooled approximately 8–10 DRG belonging to the same animal. DRGs were randomly collected along the complete spinal column and regularly included the lumbar DRG from both sides. We then performed RNA-seq on samples collected from three different animals in each experimental group. Therefore, our data originated from n = 3 independent biological replicates for each group analyzed.

Neonatal and adult DRG and sciatic nerve total RNA was isolated using TRIzol reagent (Life Technology) and processed according to the manufacturer’s instructions. Quality of the extracted RNAs was assessed using an RNA 6000 Nano Kit on an Agilent 2100 Bioanalyzer (Agilent Technologies). Libraries were prepared using the TruSeq Stranded mRNA Sample Prep Kit (Illumina), following the manufacturer’s instructions. Sequencing of pooled dorsal root ganglia and peripheral nerves collected from young adult CGM, GF, and OMM12 mice was performed using the Illumina NextSeq 500 platform. Sequencing of pooled dorsal root ganglia and peripheral nerves collected from neonatal CGM, GF, and OMM12 mice was performed using the Illumina NextSeq 1000 platform.

After quality control using FastQC (https://www.bioinformatics.babraham.ac.uk/projects/fastqc/), raw reads were aligned to the mouse reference genome (UCSC mm10/GRCm38) using STAR 2.7.1a^84^ (with parameters – outFilterMismatchNmax 999 –outFilterMismatchNoverLmax 0.04). Gene expression levels were quantified with featureCounts v1.6.3^85^ (options: -t exon -g gene_name) using the GENCODE M23 annotation. Multimapped reads were excluded from quantification. Gene expression counts were analyzed using the edgeR package.^86^ After low expression gene filtration (1 count per million (CPM) in less than 3 samples), normalization factors were calculated using the trimmed mean of M-values (TMM) method (implemented in the calcNormFactors function). Differential expression analysis was performed by fitting a GLM to all groups and performing the LF test for pairwise comparisons. Genes were considered to be significantly differentially expressed when | log_2_FC |>1 and *p* < 0.01 in each comparison. Gene set enrichment analysis was conducted using GSEA software^87^ on the log_2_FC*-logPval ranked genes. Gene expression heatmaps were generated using the ComplexHeatmap R package, scaling logCPM values as Z-scores across samples.^88^

Proteins connected to NRG1 activity were manually collected, and their functional association was retrieved from the STRING database^89^ to obtain a network. Cytoscape^90^ was used for network visualization using logFC*-logPval to characterize the nodes.

### Quantitative real time PCR (qRT-PCR) analysis of DRG and sciatic nerve RNA, and presence of OMM12 members in fecal DNA of OMM12 and CGM communities

Total RNA (500 ng) extracted from neonatal and adult DRG and sciatic nerve was reverse-transcribed and analyzed as previously described.^91^ cDNA products were analyzed by qRT-PCR using 1X iTaq Universal SYBR Green Supermix (Bio-Rad) and 300 nM forward and reverse primers in an ABI Prism 7300 (Applied Biosystems, Life Technologies Europe BV, Monza, Italy) detection system to quantify *Nrg1* expression. Data were calibrated to adult CGM samples and normalized to TATA-box-binding protein (*Tbp*) expression. *Nrg1* and *Tbp* primer sequences used are listed in Tab. S2. Neonatal samples *n* = 4 animals; young adult samples *n* = 3 animals.

The same DNA used for the shallow shot metagenomics was used for detection of OMM12 members in fecal samples from OMM12 and CGM community. The absolute detection of 16S rRNA gene copy numbers of each OMM12 member was performed using TaqMan assay specific primer/probe set described by Brugiroux et al.^11^ and Taqman® Universal Master Mix according to the manufacturer’s instruction in the QuantStudio 6 Flex Real-Time PCR System.

### Histomorphometry and electron microscopy analysis

Median nerves were collected from neonatal and young adult CGM, OMM12, and GF mice and analyzed according to an established protocol.^92^ Briefly, 5 mm long nerve samples were fixed using Karnovsky solution (2% paraformaldehyde, 2.5% glutaraldehyde in 0.2 M sodium cacodylate buffer, pH 7.3, 24 h), washed (0.1 M sodium cacodylate, 7.5% sucrose), and post–fixed (1% osmium tetroxide for 1.5 h).

Stereological analysis of the median nerve was performed as described previously^93^ using a light microscope (Olympus Europa SE & Co. KG, Hamburg, Germany) equipped with a prior controller (MBF Bioscience, Williston, VT, USA) and Stereo Investigator software, version 2021.1.1 (MBF Bioscience, Williston, VT, USA). Two sections of each sample were selected randomly to determine the cross-sectional area (20× magnification) and the number of myelinated fibers (100× magnification) and to calculate the nerve fiber density using the optical fractionator. The parameters were set as follows: grid size x = 60 µm, y = 60 µm, counting frame size x = 20 µm, and y = 20 µm (version 1.48; National Institutes of Health, Bethesda, USA).

Quantitative analysis of unmyelinated fibers was performed on ultrathin sections (70-nm-thick) analyzed using a JEM-1010 transmission electron microscope (JEOL, Tokyo, Japan) equipped with a Mega-View-III digital camera and a Soft-Imaging-System (SIS, Münster, Germany). Analysis was performed on samples from three randomly chosen animals per group; from each sample, approximately 50 Remak bundles were randomly chosen and unmyelinated axons were measured (a total of approximately 400 unmyelinated axons per group were analyzed).

### Quantitative real time PCR (qRT-PCR) in murine muscles

Whole frozen tibialis anterior and soleus muscles were ground in liquid nitrogen using a pestle and mortar. Total RNA was isolated using the TRIzol reagent (Invitrogen) and processed according to the manufacturer’s instructions. The RNA concentration and purity were assessed using a NanoDrop2000c spectrophotometer (Thermo Fisher Scientific). Reverse transcription was performed with the High-Capacity cDNA Reverse Transcription kit (Applied Biosystems) according to manufacturer’s instructions and cDNA products were analyzed by quantitative real-time PCR on a QuantStudio™5 Real-Time PCR System, 384-well (Thermo Fisher Scientific) using the 5× HOT FIREPol^Ⓡ^EvaGreen^Ⓡ^ qPCR Supermix (Solis BioDyne). Primer sequences are listed in Suppl. Table S2. The data were normalized to Ribosomal Protein L7 Like 1 (*Rpl7l1*) expression levels.

### Western blotting

Sciatic nerve total proteins were extracted using TRIzol reagent (Life Technology) after RNA extraction according to the manufacturer’s instructions. In the final passage, the protein pellet was resuspended in boiling Laemli buffer [2.5% sodium dodecyl sulfate (SDS), 0.125 M Tris – HCl pH 6.8]. Proteins (40 μg/sample) were resolved by 8% SDS – PAGE and blotted on supported nitrocellulose membrane (#1620094, Bio-Rad). Membranes were saturated for 1 h in 5% nonfat dry milk in TBS-T and incubated overnight at 4°C with primary antibodies diluted in 5% BSA TBS-T. Primary antibodies used were as follows: rabbit anti-NRG1 type III (1:1000, Chemicon International, USA, #AB5551); rabbit anti-ErbB3 (1:1000; Cell Signaling Technology, #12708); mouse anti-β-actin (1:4000; Sigma, Germany, #A5316); mouse anti-vinculin (1:2000, Sigma-Aldrich, #V9131); secondary antibodies used were HRP-linked anti-rabbit (Cell Signaling Technology, #7074) and anti-mouse (Cell Signaling Technology, #7076), both diluted 1:15000 in 5% nonfat dry milk in TBS-T. Bands were detected using ECL substrate (#170–5061, Biorad), collected with Chemidoc, quantified with Image Lab Software (Bio-Rad, California, USA), and their intensity was normalized to the geometric average of the housekeeping genes, actin and GAPDH.

Frozen tibialis anterior muscles were pulverized by using pestle and mortar in liquid nitrogen and lysed in the extraction buffer (50 mM Tris HCL pH 7.5, 150 mM NaCl, 1 mM EDTA, 10% glycerol; 0.5 mM DTT, 2% (sodium dodecyl sulfate (SDS), 1% Triton X-100) supplemented with phosphatase inhibitors (Cocktail II and III, Sigma) and protease inhibitors (complete EDTA free, Roche). SDS-PAGE of protein lysates (20 μg) was carried out in 12% or 4–12% gradient polyacrylamide NuPAGE^™^ Bis-Tris Gels (Invitrogen) and electrotransferred onto polyvinylidene difluoride (PVDF) Transfer Membranes (Thermo Scientific). Membranes were saturated for 1 h in 5% nonfat dry milk or in 5% Bovine Serum Albumin (BSA) in Tris-Buffered Saline 0.1% Tween 20 (TBS-T) and incubated overnight at 4°C with the following primary antibodies diluted in 2.5% nonfat dry milk or for phosphorylated forms in 5% BSA in TBS-T: guinea pig anti-p62/SQSTM1 (1:1000, Progen, GP62-C); mouse anti-vinculin (1:1000, Sigma-Aldrich, #V4505); rabbit anti-LC3B (1:1000, Thermo Fisher Scientific, PA1–16930); rabbit anti-AMPKα (1:1000, Cell Signaling Technology, #2532); rabbit anti-phospho-AMPKα (Thr172) (1:1000, Cell Signaling Technology, #4188); rabbit anti-phospho-S6 Ribosomal Protein (Ser240/244) (1:1000, Cell Signaling Technology, #5364); rabbit anti-S6 Ribosomal Protein (1:1000, Cell Signaling Technology, #2217). Horseradish peroxidase (HRP)-conjugated secondary antibodies (1:2000; Bethyl Laboratories) were used in 2.5% milk in TBS-T. Detection was accomplished by chemiluminescence using the WesternBright^™^ ECL HRP substrate kit (Advansta) and Amersham ImageQuant^™^ 800 (Cytiva) camera system. Densitometric quantification was performed using Fiji software. Proteins were normalized to the loading controls and then to the mean value of the control condition (CGM animals).

### Muscle histology and morphometric analysis

Tibialis anterior muscles were fixed in 4% paraformaldehyde in phosphate-buffered saline (PBS) overnight at 4°C, then dehydrated in increasing ethanol series, cleared with toluene, and embedded in paraffin. 7-μm-thick cross-sections were cut on a microtome, collected on glass slides, and stored at room temperature until use. Samples were dewaxed with xylene, rehydrated in a decreasing ethanol series, and stained with Wheat Germ Agglutinin Alexa Fluor^TM^ 488 conjugate (2 μg/mL, Invitrogen) and Hoechst 33,342 (1 μg/mL, Invitrogen) in PBS for 20 min at room temperature. Slides were washed three times with PBS and mounted in 80% glycerol-PBS. The entire muscle sections were imaged at 10× magnification using a Leica Stellaris 5 confocal microscope coupled with Leica Application Suite X (LASX) software. Cross-sectional area and minimum Feret diameter measurements were carried out using the MATLAB application SMASH.^94^ Filtering parameters were chosen to optimize the segmentation and filtering procedures according to animal age and were maintained constant for all analyses. Incorrect selections were manually corrected. Centrally nucleated fibers and the total number of myofibers per tibialis anterior section were counted manually using the Cell Counter plugin of the Fiji software.^95^

### Whole-mount staining on diaphragms and NMJ morphometric analysis

Hemidiaphragms were fixed with 4% paraformaldehyde in PBS for 1 h at room temperature, then incubated overnight at 4°C with α-bungarotoxin (αBTX) Alexa Fluor^TM^ 555 conjugated (1:1000, Invitrogen) in a blocking solution (4% BSA, 0.5% Triton X-100). Samples were finally extensively washed in TBS and mounted in 80% glycerol-PBS before imaging using a Leica Stellaris 5 confocal microscope with the Leica Application Suite X (LASX) software. Z-stacks with a 1-μm interval of single *en face* NMJs were taken with a 63× oil immersion objective, 2.5 × zoom and 512×512 frame size. For each sample, at least 20 NMJs were randomly acquired from the entire muscle. NMJ morphometry was analyzed using maximum intensity Z-stack projections, Fiji software, and the BinaryConnectivity plugin (https://blog.bham.ac.uk/intellimic/glandini-software/) combined with the NMJ-morph/aNMJ-morph workflow.^28,96^ Five morphological postsynaptic variables were quantified using this methodology, including AChR area, AChR perimeter, endplate area, endplate perimeter, and compactness [defined as AChRarea/endplate area) x100]. The endplate area consists of postsynaptic staining and all areas devoid of α-BTX within the perimeter of staining. The AChR area exclusively represents the area of staining (excluding α-BTX-negative areas). The perimeters followed the same procedure. The number of AChR fragments per NMJ was determined by visual inspection of the 3D projections using the Fiji software. The fragmentation index was calculated as previously described [1-(1/number of AChR clusters)],^28^ where an index of zero reflects an intact, pretzel-shaped NMJ, and an index that tends to one is representative of a highly fragmented NMJ. In neonatal muscles, Z-stacks with a 6-μm interval were also acquired with a 10× dry objective, a 1.5 × zoom, and a 1024 × 1024 frame size for endplate bandwidth measurements. For each sample, five to seven random fields were imaged across all muscles.

## Statistical analysis

Data are expressed as the mean ± SEM. Statistical analyses were performed using GraphPad Prism Version 8 (GraphPad Software, San Diego, CA, USA). Normal distribution was tested using the Shapiro–Wilk test. For parametric data, the statistical significance was determined by one-way ANOVA followed by Tukey’s multiple comparisons *post-hoc* test or by unpaired two-tailed Student’s t-test, as indicated. Non-parametric data were subjected to the Kruskal–Wallis test followed by Dunn’s multiple comparisons *post-hoc* test or Mann–Whitney test, as indicated. *p* value ≤ 0.05 were considered significant; *n* corresponds to the number of mice investigated per group, except where indicated otherwise.

## Data and materials availability

All data needed to evaluate the conclusions in the paper are presented in the paper and/or the Supplementary Materials. Raw data are available upon request. All next-generation sequencing data generated in this study were deposited in the Gene Expression Omnibus (GEO) repository GSE210649 (https://www.ncbi.nlm.nih.gov/geo/query/acc.cgi?acc=GSE210649.) and in the European Nucleotide Archive (ENA) at EMBL-EBI under accession number PRJEB72437 (https://www.ebi.ac.uk/ena/browser/view/PRJEB72437).

## Supplementary Material

Revised Supplementary material 20240412_.docx
