# Supplementary material for: Gut microbiota depletion delays somatic peripheral nerve development and impairs neuromuscular junction maturation
Source: Gut Microbes. 2024 Jun 7;16(1):2363015. doi: 10.1080/19490976.2024.2363015 (PMC11164225; doi:10.1080/19490976.2024.2363015)

**Supplementary figures and Tables**

**
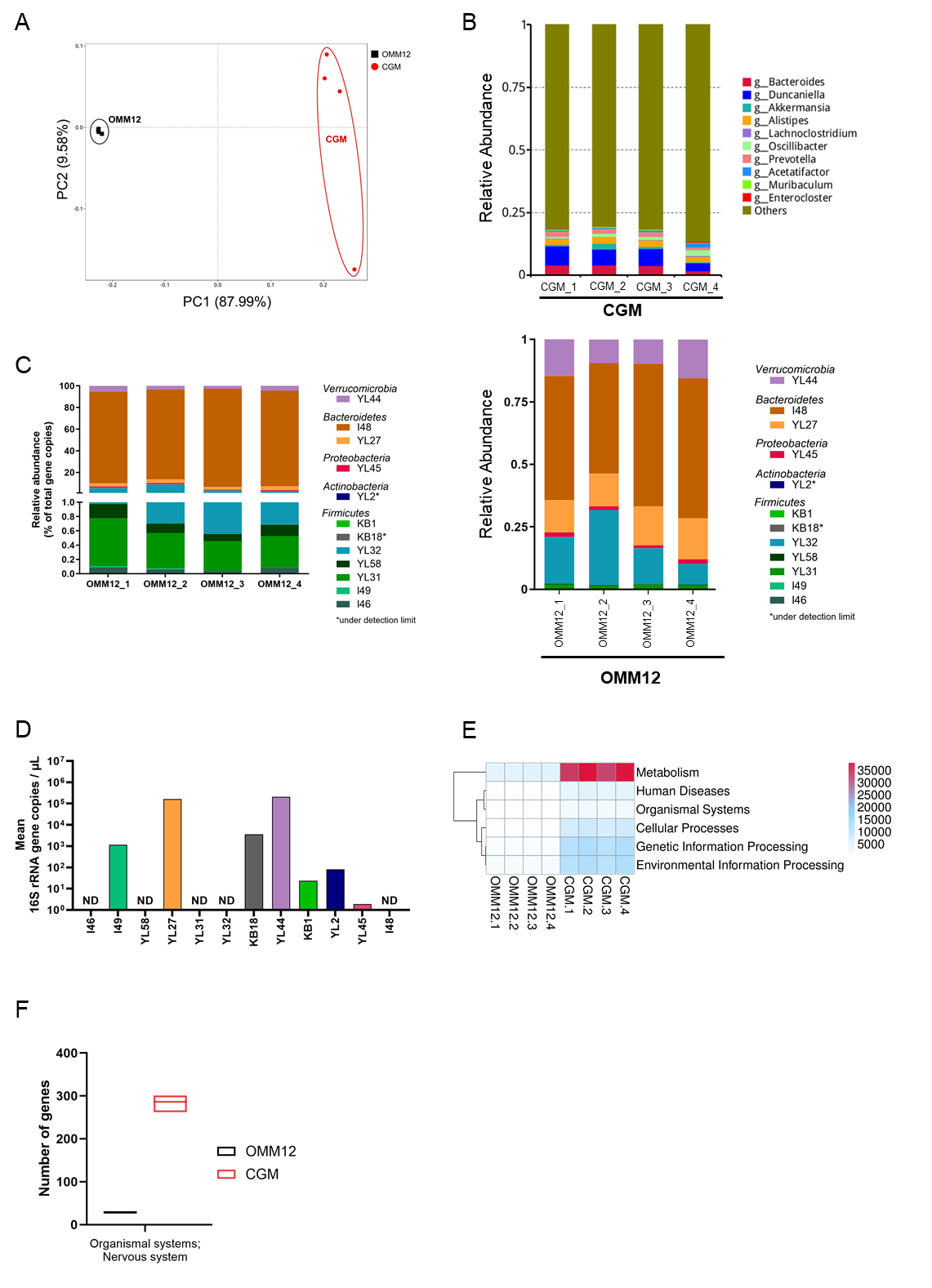
**

**Figure S1: Profiling of OMM12 and CGM communities.** **(A)** Principal coordinates analysis (PCoA) at species level. Principal coordinates are plotted to visualize the microbial community compositional differences between the CGM and OMM12 groups as well as the differences within the OMM12 and CGM group. Each point represents a single sample (one animal, n=4). The distance between points represents compositional differences between the samples. OMM12 samples are shown in black and CGM samples in red. The distance matrix is showing different microbial composition between OMM12 and CGM samples with higher compositional variation within the CGM group. **(B-C)** Relative abundance of OMM12 and CGM communities. **(B)** Based on the abundance table of genus taxonomic level, the top 10 taxa were shown for CGM community. All other taxa and unclassified data were set as “Others”. Using additional bioinformatic analysis raw sequences from OMM12 samples were aligned to the genomes of OMM12 representatives to annotate sequences to the respective bacterial genome. Unclassified sequences (0,2%) were excluded from the analysis. All classified bacterial sequences belonged to OMM12 community representatives. No reads for KB18 and YL2 were detected. No distinct taxa within the OMM12 group have been identified that would point to the group contamination. Bar charts show the relative taxonomy abundance of each fecal sample. **(C)** OMM12 community composition determined by strain-specific qPCR assay. Relative abundance of individual OMM12 strains was shown as percentage of collective 16S rRNA gene copy numbers of all OMM12 strains. KB18 and YL2 were not detected with the assay. Bar charts show the relative taxonomy abundance of each fecal sample. **(D)** Presence of OMM12 species in CGM community. Using OMM12 strain-specific 16S rRNA gene copy number qPCR analysis, the presence of OMM12 representatives in the CGM community was determined (n=4). Seven OMM12 species were confirmed in the CGM community (I49, YL27, KB18, YL44, KB1, YL2, and YL45). Bar charts show mean value of 16S rRNA copy numbers for each of OMM12 species (ND – not detected). **(E-F)** Community functional annotation based on KEGG database. **(E)** Heatmap analysis of annotated genes at level 1. The heatmap shows the difference in annotated genes between OMM12 and CGM community. The main difference between OMM12 and CGM community is attributed to the genes annotated to metabolic pathways. **(F)** Absolute abundance of KEGG annotation at level 2 for neural networks. The difference in annotated genes annotated to pathways of nervous system between OMM12 and CGM community.

**
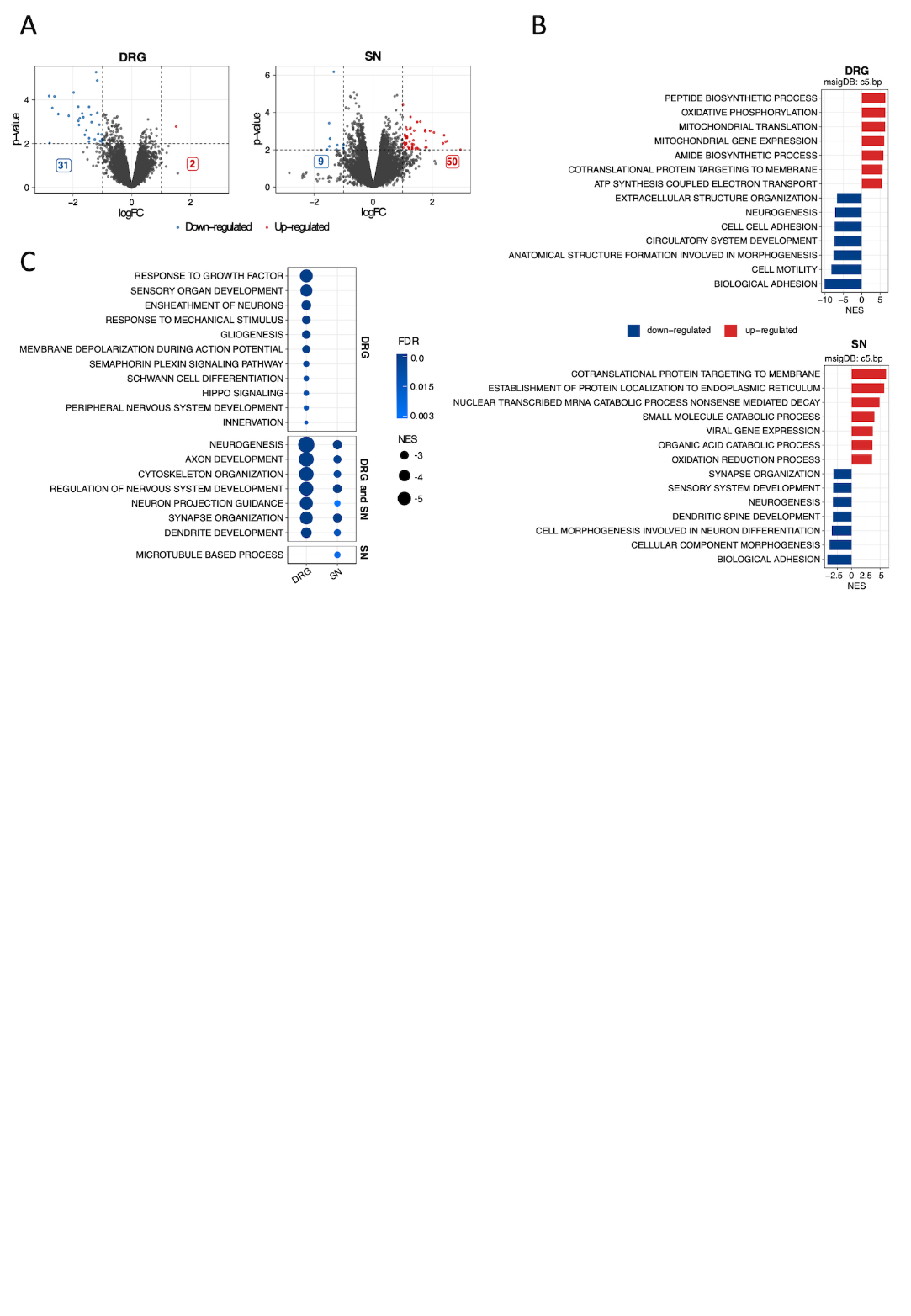
**

**Figure S2:** **Transcriptomic analysis of neonatal OMM12 compared to CGM dorsal root ganglia (DRG) and sciatic nerves.** (**A**) Volcano plots showing differentially expressed genes in neonatal DRG and sciatic nerves. Genes with a log_2_FC >1 or <1 and with -log10p-value >2 are highlighted in red and blue respectively. (**B**) Top 7 positively correlated (red) and negatively correlated (blue) with OMM12 phenotype GO biological processes (FDR <0.01) from GSEA of neonatal DRG and sciatic nerves. The reported value is the Normalised Enrichment Score (NES). (**C**) Dot plot showing a list of selected negatively enriched GO gene sets related to peripheral nervous system development, maturation and function. Dot size relates to NES value; dot colour represents the FDR. The biological processes are divided into three groups: significantly correlated in DRG only (top), sciatic nerve only (bottom) and both (centre). (FDR: false discovery rate; GO: gene ontology; SN: sciatic nerve).


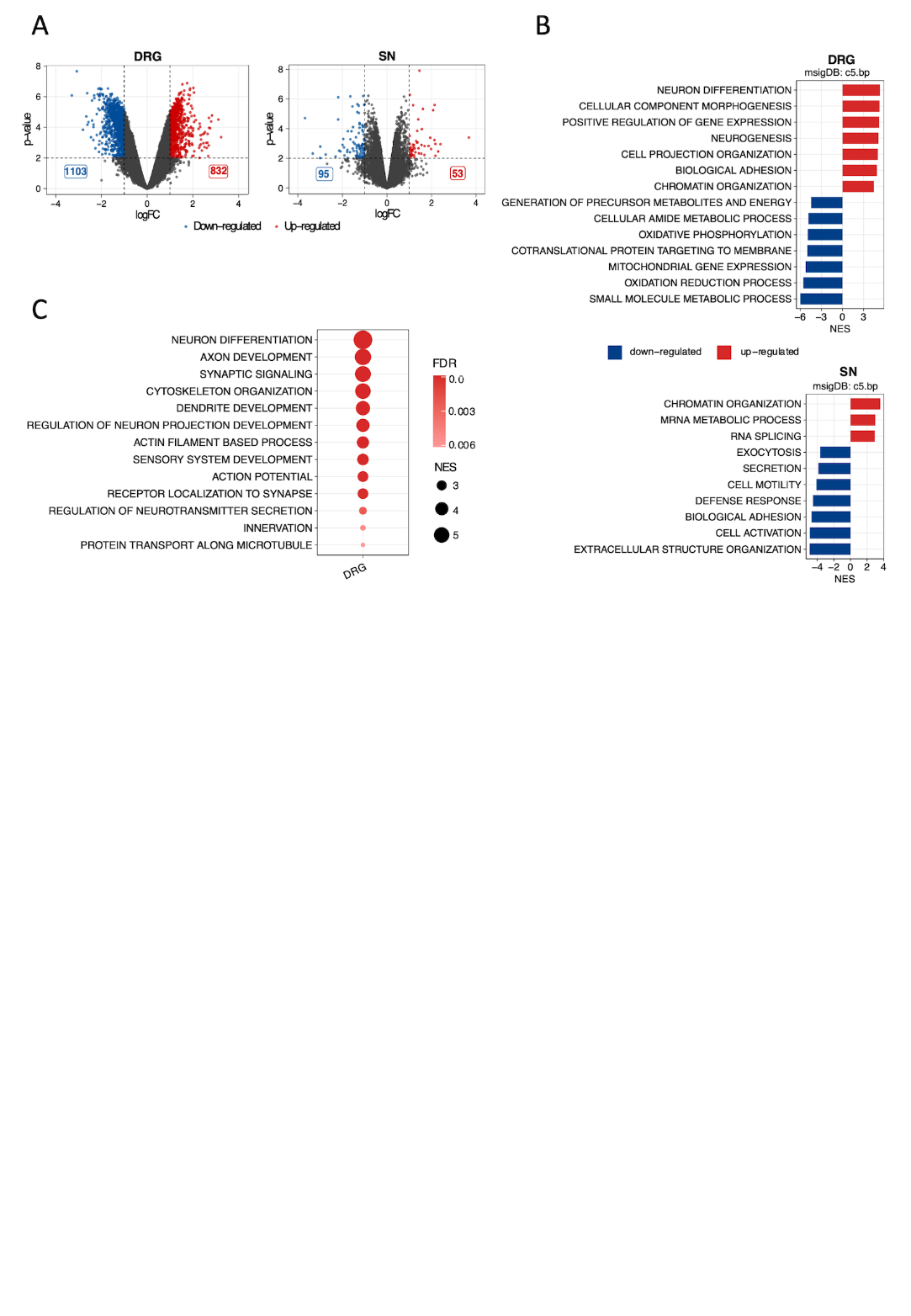


**Figure S3:** **Transcriptomic analysis of adult OMM12 compared to CGM dorsal root ganglia (DRG) and sciatic nerves.** (**A**) Volcano plots showing differentially expressed genes in DRG and sciatic nerves. Genes with a log_2_FC >1 or <1 and with -log10p-value >2 are highlighted in red and blue respectively. (**B**) Top 7 positively correlated (red) and negatively correlated (blue) with OMM12 phenotype GO gene sets (FDR <0.01) from GSEA of neonatal DRG and sciatic nerves. The reported value is the Normalised Enrichment Score (NES). (**C**) Dot plot showing a list of selected positively enriched GO gene sets related to peripheral nervous system development, maturation and function. Dot size relates to NES value; dot colour represents the FDR. (FDR: false discovery rate; GO: gene ontology; SN: sciatic nerve).

**
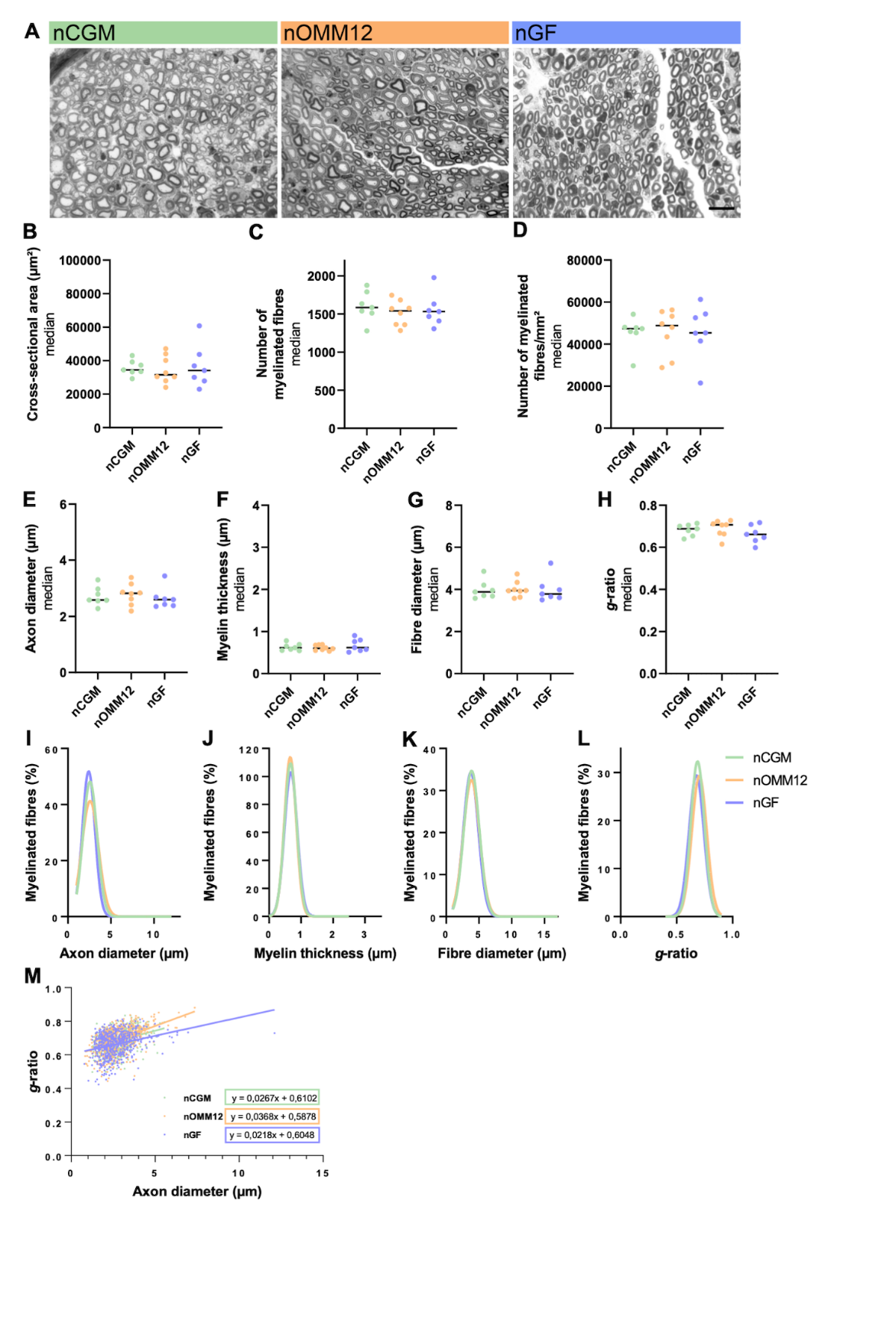
**

**Figure S4: Histomorphometrical analysis of the neonatal median nerves.** (**A**) Representative photomicrographs of toluidine blue stained semi-thin cross-sections of the neonatal median nerve that have been analysed regarding stereological (**B-D**) and morphometrical (**E-H**) parameters, including the cross-sectional area (**B**), total number of myelinated fibres (**C**), nerve fibre density (myelinated fibres/mm²) (**D**), axon diameter (**E**), myelin thickness (**F**), fibre diameter (**G**), and g-ratio (**H**). Scatter plots depict the median of shown individual values. nCGM *n* = 7, nOMM12 *n* = 8, and nGF *n* = 7 animals. Scale bar: 10 µm. Regression curves (Gaussian equation, not given) of percentile distribution for axon diameter (**I**), myelin thickness (**J**), fibre diameter (**K**), and g-ratio (**L**) and a scatter plot showing the g-ratio of individual myelinated axons as a function of the respective axon diameter (**M**) were selected to present morphometrical differences in further detail. Equation of linear regressions are given. nCGM *n* = 560, nOMM12 *n* = 640, and nGF *n* = 560 axons. Please notice that y-axis scaling varies. All data was subjected to non-parametric Kruskall-Wallis test followed by Dunn's multiple comparisons post-hoc test.

**
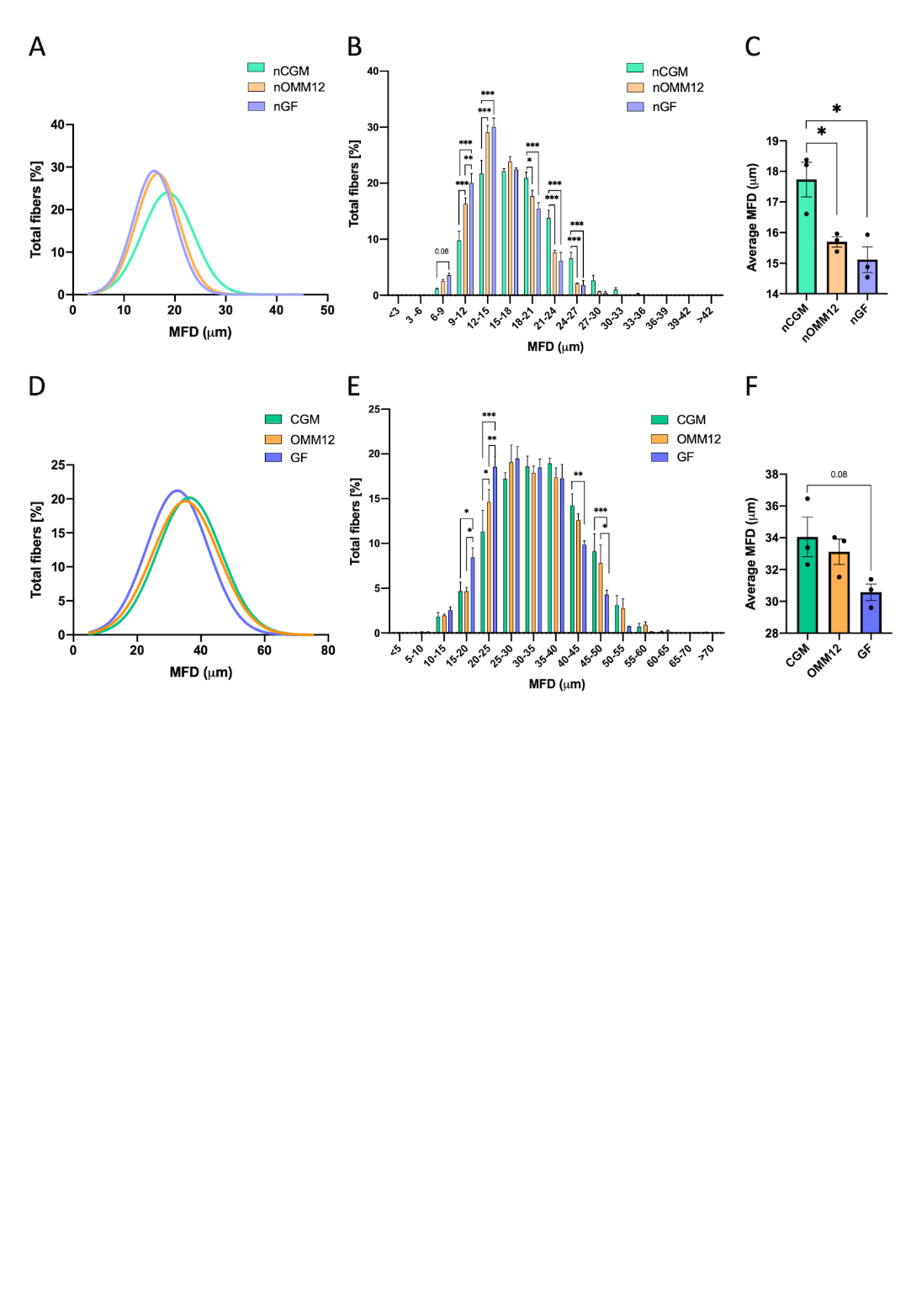
**

**Figure S5.** Morphometric analysis of muscle fibres in neonatal and adult muscles. (**A**) Graphical representation of minimum Feret’s diameter (MFD) distribution among myofibers in tibialis anterior sections from neonatal nCGM, nOMM12 and nGF mice. The curves were fitted to data using non-linear regression (Gaussian). (**B**) MFD distribution among myofibers for the three hygiene status groups of neonatal animals. The comparison between the groups is provided for each class of MFD distribution. Two-way ANOVA with post-hoc Tukey’s test for multiple comparisons. (**C**) Average values of MFD were evaluated for nCGM, nOMM12 and nGF muscles. Normal distribution was tested using the Shapiro-Wilk test; one-way ANOVA with post-hoc Tukey’s test for multiple comparisons; n = 3 mice, each group. (**D**) Graphical representation of MFD distribution among myofibers in tibialis anterior sections from young adult CGM, OMM12 and GF mice. The curves were fitted to data using non-linear regression (Gaussian). (**E**) MFD distribution among myofibers for the three hygiene status groups of young adult animals. The comparison between the groups is provided for each class of MFD distribution. Two-way ANOVA with post-hoc Tukey’s test for multiple comparisons. (**F**) Average values of MFD were evaluated for CGM, OMM12 and GF muscles. Normal distribution was tested using the Shapiro-Wilk test; one-way ANOVA with post-hoc Tukey’s test for multiple comparisons; n = 3 mice, each group. Bar graphs depict the mean ± SEM; * P ≤ 0.05, ** P ≤ 0.01, *** P <0.001.


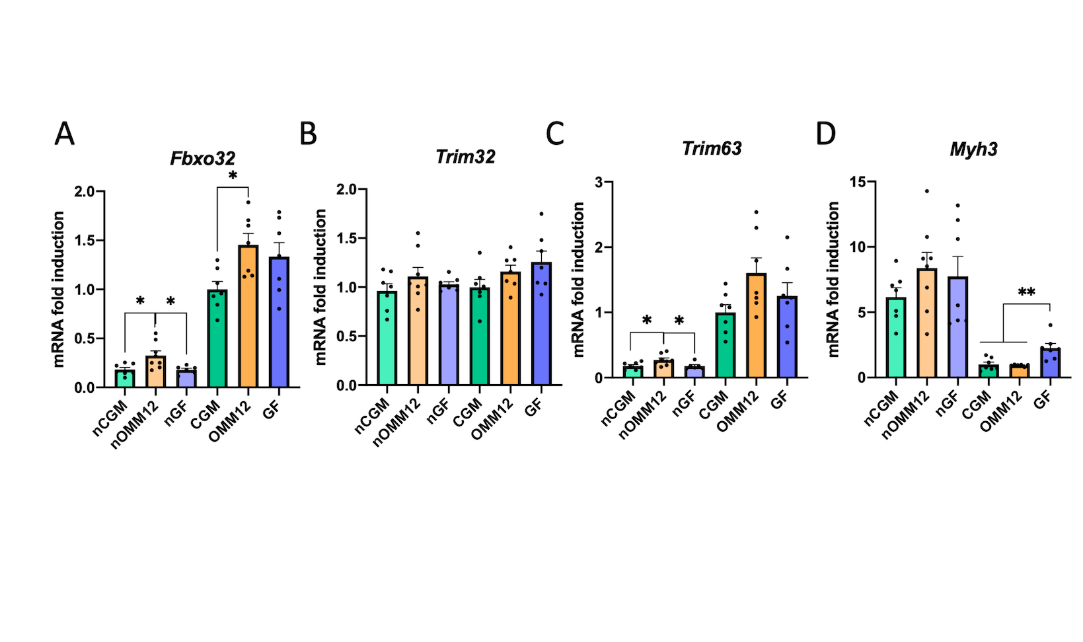


**Figure S6.** **Expression of atrogenes and *Myh3* in soleus muscles**. qRT-PCR analysis for transcripts coding for the atrogenes *Fbxo32* (**A**) *Trim32* (**B**) and *Trim63* (**C**) and the embryonic *Myh3* in mRNA extracts from soleus muscles of GF, OMM12 and CGM newborn and young adult mice. One-way ANOVA with post-hoc Tukey’s test for multiple comparisons; normal distribution tested using the Shapiro-Wilk test. Bar graphs depict the mean ± SEM; * *P* ≤ 0.05, ** *P* ≤ 0.01.


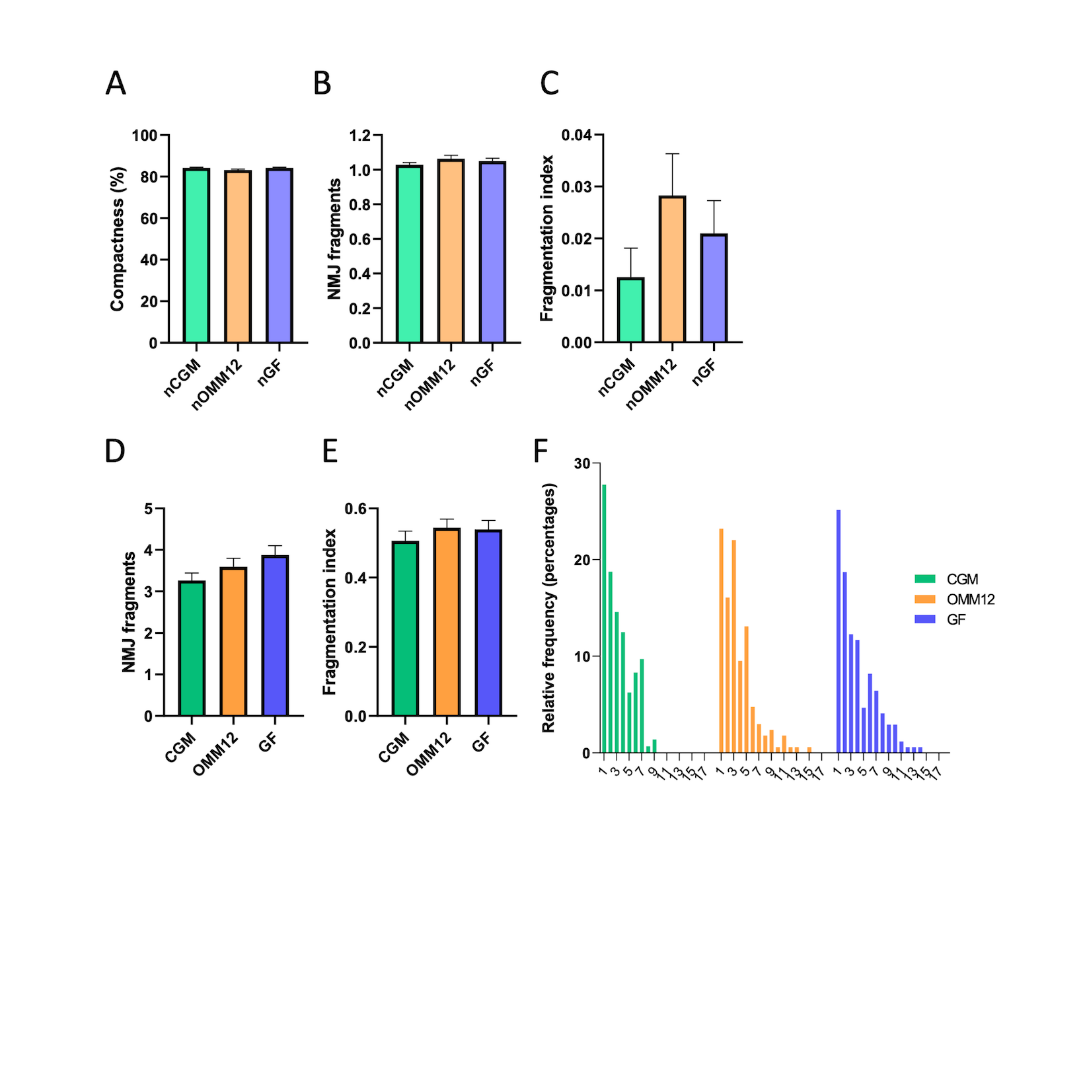


**Figure S7**. **Analysis of NMJ morphometry and fragmentation in neonatal and adult mice.** (**A-C**) Quantitative analysis of postsynaptic variables from diaphragm muscles of CGM, OMM12 and GF neonatal mice showing compactness (**A**), mean number of fragments (**B**), and fragmentation index (**C**). Normal distribution was tested using the Shapiro-Wilk test; Kruskal-Wallis test with post-hoc Dunn’s test for multiple comparisons; NMJs n = 213 for CGM; n = 221 for OMM12; n = 282 for GF. (**D-E**) Quantitative analysis of postsynaptic variables from diaphragm muscles of CGM, OMM12 and GF young adult mice showing mean number of fragments (**E**), and fragmentation index (**F**). (**H**) Relative frequency distribution of NMJ fragments in adult mice. NMJs n = 144 for CGM; n = 168 for OMM12; n = 171 for GF. Data are shown as mean ± SEM.

**Table S1.** Statistical analysis comparing histomorphometrical data about myelinated fibers obtained from neonatal and young adult mice.
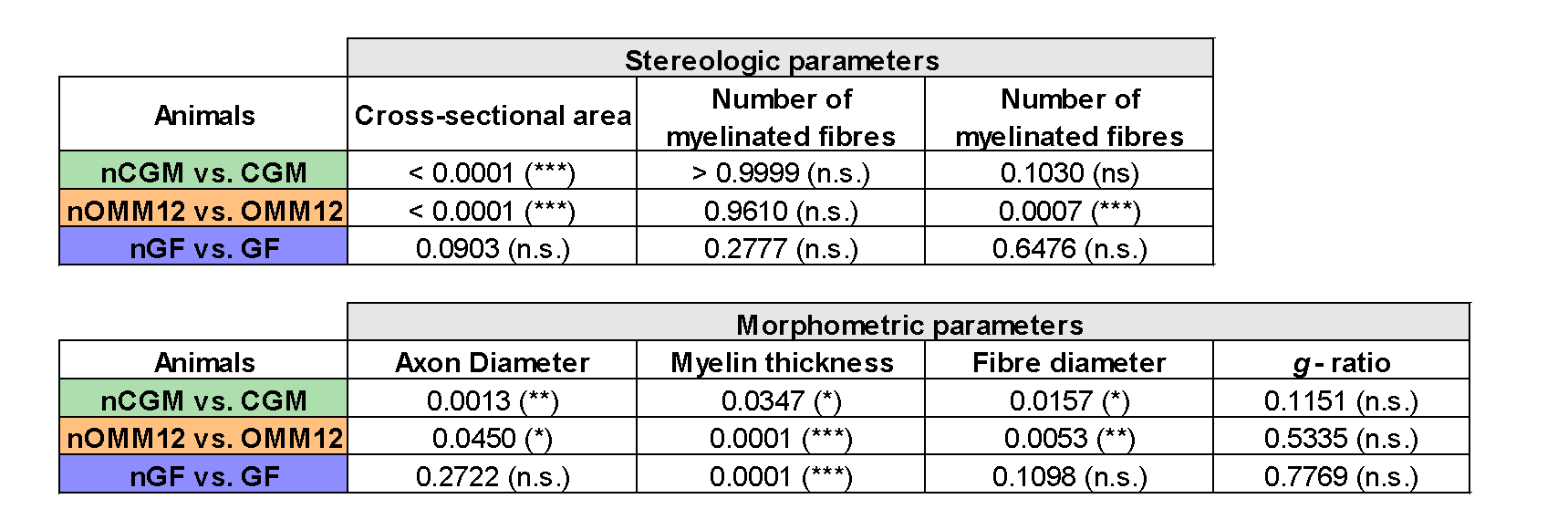


**Table S2.** List of primers used for qRT-PCR.
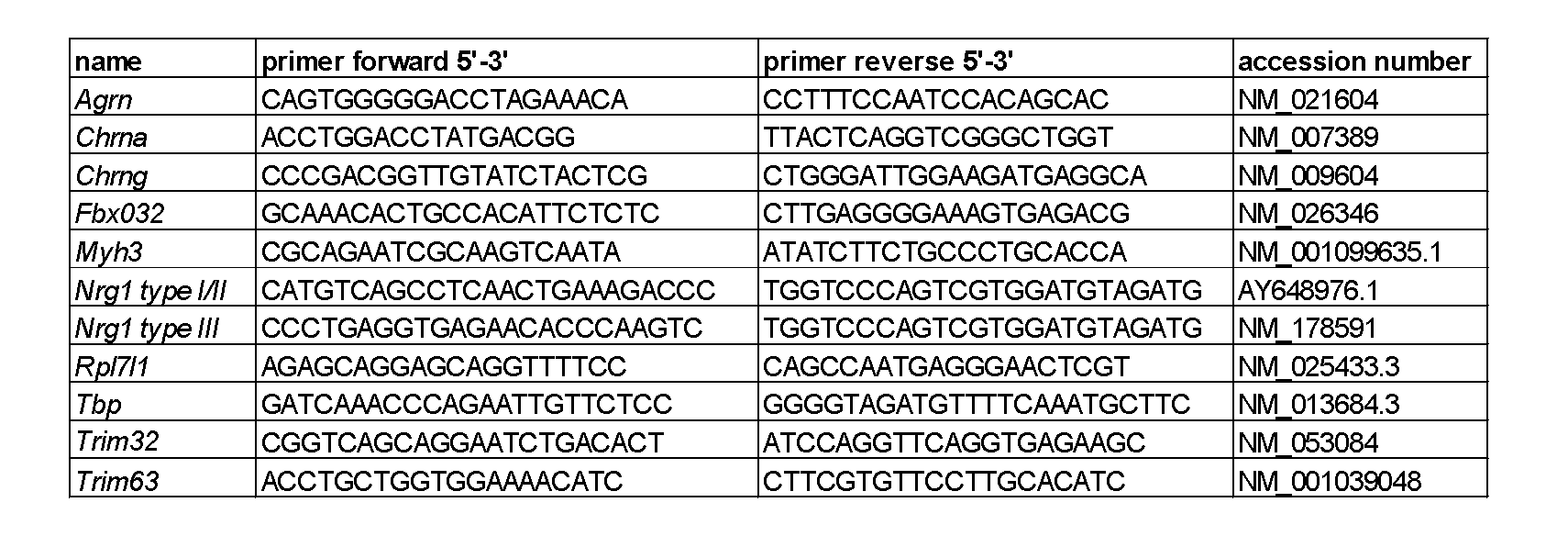

Supplement: Revised Supplementary material 20240412_.docx [file KGMI_A_2363015_SM9921.docx]
